# Supplementary material for: Spatial and temporal distribution of thyroid cancer incidence in China: An ecological study based on a national registry
Source: PLoS One. 2026 Mar 20;21(3):e0341734. doi: 10.1371/journal.pone.0341734 (PMC13004386; doi:10.1371/journal.pone.0341734)
Supplement: S1 File — (DOCX) [file pone.0341734.s001.docx]

**Spatial and temporal distribution of thyroid cancer incidence in China: An ecological study based on a national registry**

**Contents**

**S1 Table……………………………………………………………………………………. 1**

**S2 Table……………………………………………………………………………………. 3**

**S3 Table……………………………………………………………………………………. 5**

**S4 Table……………………………………………………………………………………. 7**

**S5 Table……………………………………………………………………………………. 9**

**S1 Table. Coverage population and age-specific incidence rate of thyroid cancer in cancer registration areas of China, 2005-2018**

| Year | Nation | 0- | 1-4 | 5-9 | 10-14 | 15-19 | 20-24 | 25-29 | 30-34 | 35-39 | 40-44 | 45-49 | 50-54 | 55-59 | 60-64 | 65-69 | 70-74 | 75-79 | 80-84 | 85+ |
| --- | --- | --- | --- | --- | --- | --- | --- | --- | --- | --- | --- | --- | --- | --- | --- | --- | --- | --- | --- | --- |
| 2005 | Population | 328711 | 1499272 | 2496876 | 3308064 | 4262016 | 4736635 | 4186010 | 4567224 | 4658126 | 5008480 | 4842373 | 4093564 | 2840819 | 2109422 | 2049628 | 1781580 | 1164872 | 628448 | 361567 |
| 2005 | Thyroid cancer | 0.00 | 0.00 | 0.08 | 0.27 | 0.73 | 2.03 | 2.99 | 3.31 | 4.32 | 6.75 | 6.96 | 8.16 | 7.22 | 7.25 | 6.88 | 6.17 | 6.7 | 5.25 | 4.7 |
| 2006 | Population | 366806 | 1664935 | 2554453 | 3463714 | 4346208 | 4986761 | 4689395 | 4914363 | 5312157 | 5491492 | 5098023 | 4605257 | 3195320 | 2369247 | 2184071 | 1951450 | 1286489 | 692922 | 394259 |
| 2006 | Thyroid cancer | 0.00 | 0.00 | 0.12 | 0.29 | 0.76 | 2.27 | 3.88 | 4.42 | 5.61 | 6.72 | 7.73 | 10.4 | 8.67 | 6.63 | 6.55 | 8.4 | 5.6 | 6.93 | 4.57 |
| 2007 | Population | 406298 | 1692059 | 2533898 | 3380370 | 4330107 | 5241847 | 4818883 | 4701146 | 5301698 | 5365826 | 4920675 | 4694874 | 3352967 | 2472038 | 2158312 | 1967923 | 1316812 | 731771 | 421807 |
| 2007 | Thyroid cancer | 0.00 | 0.06 | 0.24 | 0.15 | 1.22 | 2.06 | 3.78 | 5.08 | 5.55 | 7.47 | 8.78 | 11.48 | 8.74 | 7.44 | 7.97 | 7.57 | 6.3 | 6.97 | 4.74 |
| 2008 | Population | 435222 | 1873291 | 2562894 | 3429471 | 4303883 | 5632792 | 5472349 | 5029723 | 5757160 | 5652071 | 5690334 | 5549035 | 4144899 | 2983245 | 2334053 | 2213476 | 1616957 | 911879 | 546050 |
| 2008 | Thyroid cancer | 0.00 | 0.05 | 0.16 | 0.15 | 1.18 | 2.57 | 5.01 | 5.71 | 7.47 | 8.63 | 11.39 | 13.1 | 11.89 | 9.25 | 8.61 | 8.22 | 7.17 | 6.25 | 9.16 |
| 2009 | Population | 620042 | 2733157 | 3612024 | 4558667 | 5426773 | 7355399 | 7240893 | 6644560 | 7331681 | 7196705 | 7354331 | 6638912 | 5485661 | 3916364 | 2951001 | 2623699 | 1984938 | 1121727 | 673988 |
| 2009 | Thyroid cancer | 0.00 | 0.07 | 0.11 | 0.37 | 1.18 | 2.61 | 5.05 | 5.79 | 7.42 | 7.81 | 11.23 | 12.5 | 12.72 | 10.44 | 8.2 | 8.35 | 7.56 | 5.9 | 4.75 |
| 2010 | Population | 1039937 | 4478478 | 5691873 | 6632136 | 7631685 | 10402751 | 10258234 | 9674312 | 10695580 | 10837964 | 10700624 | 9271970 | 8211844 | 5800088 | 4226310 | 3660461 | 2856613 | 1626951 | 955124 |
| 2010 | Thyroid cancer | 0.00 | 0.00 | 0.05 | 0.3 | 0.93 | 2.41 | 4.78 | 6.3 | 7.05 | 7.85 | 10.64 | 12.37 | 12.07 | 9.71 | 7.83 | 6.26 | 6.13 | 4.67 | 4.19 |
| 2011 | Population | 1296974 | 5526014 | 6691064 | 7423553 | 8822941 | 11875975 | 11926174 | 11344972 | 12182268 | 12759799 | 12629340 | 10367143 | 10032914 | 7114395 | 5113489 | 4191455 | 3369063 | 1939748 | 1141190 |
| 2011 | Thyroid cancer | 0.00 | 0.02 | 0.07 | 0.23 | 0.99 | 3.06 | 6.09 | 8.5 | 9.45 | 10.82 | 13.74 | 14.68 | 13.84 | 12.48 | 9.76 | 8.33 | 5.55 | 5.93 | 4.56 |
| 2012 | Population | 1876699 | 7639169 | 8966889 | 9485568 | 11949307 | 16424691 | 16149358 | 15419192 | 15927097 | 17829944 | 17092061 | 13679936 | 14055861 | 10112526 | 7040979 | 5563404 | 4541419 | 2697064 | 1615242 |
| 2012 | Thyroid cancer | 0.00 | 0.00 | 0.08 | 0.23 | 1.21 | 3.16 | 6.42 | 9.4 | 10.56 | 13.05 | 15.47 | 16.01 | 15.42 | 13.14 | 10.71 | 7.84 | 5.9 | 4.6 | 3.9 |
| 2013 | Population | 2111613 | 8951470 | 10939492 | 10644355 | 13241576 | 17987676 | 18225832 | 17225402 | 17688823 | 20262762 | 19593179 | 15942076 | 16276706 | 12229639 | 8413853 | 6396462 | 5227037 | 3176953 | 1963784 |
| 2013 | Thyroid cancer | 0.00 | 0.03 | 0.05 | 0.23 | 1.02 | 3.24 | 7.48 | 11.85 | 13.02 | 15.11 | 16.65 | 20.18 | 18.1 | 15.75 | 11.16 | 8.68 | 7.52 | 5.13 | 4.53 |
| 2014 | Population | 2814044 | 11790643 | 14158817 | 13477475 | 16278953 | 22270337 | 23654798 | 21777508 | 22178903 | 25154963 | 24871943 | 20860587 | 19878526 | 16141504 | 11185605 | 8198251 | 6623616 | 4220840 | 2706034 |
| 2014 | Thyroid cancer | 0.04 | 0.03 | 0.06 | 0.27 | 1.12 | 3.73 | 8.96 | 14.36 | 16.33 | 18.85 | 19.54 | 25.55 | 22.45 | 18.73 | 13.69 | 9.32 | 7.05 | 5.31 | 3.73 |
| 2015 | Population | 3019983 | 13723612 | 16269160 | 14899753 | 17255204 | 23172769 | 26646434 | 24318007 | 23980557 | 27069161 | 28165992 | 24368121 | 21118676 | 18741286 | 13102481 | 9531954 | 7359252 | 4867545 | 3305902 |
| 2015 | Thyroid cancer | 0.00 | 0.01 | 0.04 | 0.34 | 1.39 | 4.34 | 10.75 | 16.31 | 18.08 | 19.83 | 20.02 | 27.21 | 23.38 | 19.34 | 13.68 | 9.62 | 6.44 | 5.22 | 4.36 |
| 2016 | Population | 3815719 | 16507793 | 20027315 | 18146881 | 20633230 | 26580417 | 30491843 | 27908572 | 29065165 | 31514690 | 34173467 | 28879966 | 24484064 | 22818464 | 16408891 | 11537525 | 8712937 | 5872725 | 3985758 |
| 2016 | Thyroid cancer | 0.08 | 0.02 | 0.04 | 0.26 | 1.27 | 4.55 | 11.2 | 17.41 | 17.96 | 19.6 | 20.73 | 28.18 | 21.19 | 19.4 | 14.03 | 9.3 | 6.46 | 4.89 | 3.99 |
| 2017 | Population | 4653648 | 19735402 | 24034832 | 21588488 | 23266394 | 29014896 | 33333111 | 31957806 | 33496246 | 35065408 | 39300205 | 32891678 | 27318505 | 26394347 | 19479486 | 13536013 | 9963871 | 6726575 | 4580044 |
| 2017 | Thyroid cancer | 0.00 | 0.02 | 0.08 | 0.20 | 1.19 | 5.04 | 12.88 | 18.28 | 20.26 | 20.41 | 22.47 | 29.06 | 21.71 | 19.74 | 14.29 | 9.60 | 6.70 | 5.20 | 3.93 |
| 2018 | Population | 5351889 | 23931238 | 28842375 | 26591483 | 27727794 | 34515110 | 38605303 | 38663141 | 39956042 | 41246056 | 46480021 | 39175921 | 33671990 | 31384128 | 24472174 | 16823799 | 11984179 | 8101104 | 5636502 |
| 2018 | Thyroid cancer | 0.00 | 0.03 | 0.06 | 0.35 | 1.36 | 5.31 | 14.87 | 22.03 | 24.74 | 24.33 | 26.59 | 31.03 | 26.73 | 23.01 | 16.65 | 10.44 | 6.85 | 5.49 | 4.38 |

**S2 Table. Coverage population and age-specific incidence rate of thyroid cancer in urban cancer registration areas of China, 2005-2018**

| Year | Urban | 0- | 1-4 | 5-9 | 10-14 | 15-19 | 20-24 | 25-29 | 30-34 | 35-39 | 40-44 | 45-49 | 50-54 | 55-59 | 60-64 | 65-69 | 70-74 | 75-79 | 80-84 | 85+ |
| --- | --- | --- | --- | --- | --- | --- | --- | --- | --- | --- | --- | --- | --- | --- | --- | --- | --- | --- | --- | --- |
| 2005 | Population | 196647 | 942781 | 1595741 | 2118775 | 3107960 | 3716148 | 3016827 | 3250134 | 3419743 | 3907878 | 3793112 | 3177116 | 2170410 | 1579718 | 1578770 | 1418167 | 916331 | 488471 | 281640 |
| 2005 | Thyroid cancer | 0.00 | 0.00 | 0.13 | 0.33 | 0.8 | 2.39 | 3.75 | 4.09 | 5.21 | 7.93 | 8.09 | 9.66 | 8.39 | 8.61 | 7.6 | 7.26 | 6.98 | 5.94 | 6.04 |
| 2006 | Population | 246212 | 1144961 | 1769989 | 2378075 | 3303648 | 4067948 | 3640345 | 3712692 | 4144736 | 4445006 | 4123887 | 3754525 | 2590995 | 1896558 | 1766583 | 1622114 | 1061428 | 568714 | 319692 |
| 2006 | Thyroid cancer | 0.00 | 0.00 | 0.17 | 0.42 | 0.88 | 2.61 | 4.42 | 5.36 | 6.56 | 7.38 | 9.07 | 11.24 | 9.53 | 7.59 | 7.25 | 9.37 | 5.94 | 7.74 | 4.69 |
| 2007 | Population | 256582 | 1081901 | 1602218 | 2115986 | 3116729 | 4129958 | 3561238 | 3302827 | 3984412 | 4168116 | 3823936 | 3730955 | 2625116 | 1888634 | 1658869 | 1588323 | 1056345 | 581230 | 335763 |
| 2007 | Thyroid cancer | 0.00 | 0.09 | 0.37 | 0.24 | 1.35 | 2.47 | 4.77 | 6.57 | 6.7 | 8.54 | 10.07 | 13.16 | 10.17 | 8.68 | 9.22 | 8.25 | 6.82 | 7.57 | 5.36 |
| 2008 | Population | 320424 | 1390007 | 1839133 | 2376365 | 3290178 | 4664290 | 4390705 | 3807575 | 4471333 | 4458786 | 4593490 | 4562163 | 3392635 | 2384913 | 1851792 | 1818933 | 1343086 | 753584 | 449103 |
| 2008 | Thyroid cancer | 0.00 | 0.07 | 0.22 | 0.21 | 1.43 | 2.92 | 5.9 | 6.91 | 8.61 | 9.82 | 13.04 | 14.47 | 13.32 | 10.19 | 9.4 | 9.24 | 7.74 | 6.9 | 10.02 |
| 2009 | Population | 384131 | 1691678 | 2109210 | 2642449 | 3526101 | 5203648 | 5014122 | 4284268 | 4879194 | 4805714 | 5095806 | 4766326 | 3867286 | 2676971 | 1964822 | 1852949 | 1433149 | 803947 | 487238 |
| 2009 | Thyroid cancer | 0.00 | 0.06 | 0.14 | 0.53 | 1.47 | 3.29 | 6.36 | 7.66 | 8.81 | 9.99 | 14.01 | 14.94 | 15.57 | 12.36 | 9.42 | 10.2 | 8.79 | 7.09 | 5.75 |
| 2010 | Population | 595402 | 2632533 | 3255545 | 3860128 | 4565411 | 6802799 | 6983769 | 6239770 | 6843382 | 6788656 | 7063029 | 6244556 | 5491662 | 3761637 | 2700579 | 2437666 | 1953659 | 1107798 | 660018 |
| 2010 | Thyroid cancer | 0.00 | 0.00 | 0.06 | 0.34 | 1.07 | 3.1 | 6.16 | 8.41 | 9.37 | 10.05 | 13.03 | 15.68 | 15.22 | 12.12 | 9.66 | 7.22 | 7.52 | 6.05 | 5.61 |
| 2011 | Population | 688959 | 2995203 | 3574164 | 4058756 | 4822662 | 6883369 | 7481728 | 6978345 | 7275155 | 7568451 | 7754987 | 6577453 | 6412883 | 4457196 | 3094084 | 2649561 | 2211567 | 1280054 | 764644 |
| 2011 | Thyroid cancer | 0.00 | 0.00 | 0.08 | 0.17 | 1.22 | 4.23 | 8.02 | 11.26 | 12.65 | 14.14 | 17.56 | 18.75 | 17.67 | 15.97 | 12.28 | 10 | 6.24 | 6.48 | 5.49 |
| 2012 | Population | 841767 | 3392704 | 3857440 | 4082043 | 5401778 | 8120890 | 8670884 | 8227366 | 8033476 | 8927869 | 8809992 | 7386623 | 7729168 | 5380756 | 3603545 | 2942061 | 2539566 | 1542357 | 959824 |
| 2012 | Thyroid cancer | 0.00 | 0.00 | 0.05 | 0.34 | 1.76 | 4.57 | 9.63 | 13.8 | 15.34 | 18.65 | 21.21 | 22.3 | 20.93 | 18.31 | 14.76 | 9.99 | 7.48 | 5.71 | 4.58 |
| 2013 | Population | 923346 | 3905782 | 4570482 | 4298465 | 5806288 | 8703488 | 9364450 | 9071652 | 8690950 | 9907464 | 9603439 | 8401497 | 8772903 | 6357947 | 4218575 | 3266396 | 2834701 | 1774505 | 1127642 |
| 2013 | Thyroid cancer | 0.00 | 0.05 | 0.09 | 0.37 | 1.53 | 4.61 | 10.96 | 17.76 | 19.62 | 21.37 | 23.89 | 27.53 | 25.2 | 21.82 | 15.5 | 11.17 | 9.28 | 5.86 | 5.94 |
| 2014 | Population | 1275248 | 5320656 | 6113824 | 5735117 | 7421242 | 10996282 | 12064510 | 11670562 | 11256245 | 12519440 | 12252736 | 10961070 | 10631745 | 8410046 | 5702607 | 4231840 | 3584481 | 2356926 | 1557338 |
| 2014 | Thyroid cancer | 0.08 | 0.02 | 0.1 | 0.33 | 1.58 | 5.14 | 13.2 | 20.74 | 23.83 | 26.75 | 26.75 | 35.25 | 31.15 | 25.47 | 18.94 | 12.26 | 8.56 | 6.62 | 4.11 |
| 2015 | Population | 1352817 | 6032410 | 6753253 | 6174671 | 7672481 | 10931136 | 12843924 | 12617080 | 11997918 | 12952773 | 13175990 | 12160461 | 10880355 | 9368698 | 6405047 | 4638105 | 3782920 | 2606840 | 1777977 |
| 2015 | Thyroid cancer | 0.00 | 0.00 | 0.07 | 0.36 | 1.77 | 6.08 | 16 | 23.71 | 25.7 | 27.61 | 27.31 | 35.94 | 31.56 | 26.07 | 17.89 | 12.46 | 8.01 | 5.83 | 5.57 |
| 2016 | Population | 1851490 | 7766990 | 9047144 | 8169491 | 9641279 | 13124739 | 15365949 | 15194692 | 15331675 | 15878586 | 17148615 | 14778468 | 13071712 | 11910003 | 8398267 | 5908537 | 4605815 | 3209086 | 2225832 |
| 2016 | Thyroid cancer | 0.11 | 0.04 | 0.08 | 0.28 | 1.46 | 5.94 | 15.73 | 23.79 | 24.49 | 26.26 | 26.66 | 36.34 | 28.12 | 25.35 | 18.06 | 11.59 | 8.01 | 6.01 | 5.08 |
| 2017 | Population | 2253504 | 9354770 | 10924785 | 9467325 | 10298582 | 13439991 | 16221263 | 16663399 | 17135841 | 17062350 | 19186102 | 16323797 | 13891567 | 13531239 | 9734324 | 6650361 | 5032828 | 3554380 | 2519875 |
| 2017 | Thyroid cancer | 0.00 | 0.00 | 0.14 | 0.23 | 1.6 | 6.99 | 18.06 | 25.9 | 28.17 | 27.95 | 29.21 | 37.39 | 28.74 | 26.27 | 18.95 | 12.12 | 8.48 | 6.5 | 4.33 |
| 2018 | Population | 2408681 | 10546850 | 12070648 | 10897060 | 11359200 | 14658239 | 17177709 | 18467999 | 19162520 | 18676972 | 21022330 | 17586441 | 15765361 | 14871872 | 11388103 | 7730530 | 5542420 | 3904609 | 2809937 |
| 2018 | Thyroid cancer | 0.00 | 0.04 | 0.04 | 0.34 | 1.63 | 7.09 | 20.75 | 30.14 | 32.98 | 32.48 | 33.55 | 38.03 | 33.3 | 29.46 | 20.4 | 12.61 | 8.21 | 6.68 | 5.59 |

**S3 Table. Coverage population and age-specific incidence rate of thyroid cancer in **rural** cancer registration areas of China, 2005-2018**

| Year | Rural | 0- | 1-4 | 5-9 | 10-14 | 15-19 | 20-24 | 25-29 | 30-34 | 35-39 | 40-44 | 45-49 | 50-54 | 55-59 | 60-64 | 65-69 | 70-74 | 75-79 | 80-84 | 85+ |
| --- | --- | --- | --- | --- | --- | --- | --- | --- | --- | --- | --- | --- | --- | --- | --- | --- | --- | --- | --- | --- |
| 2005 | Population | 132064 | 556491 | 901135 | 1189289 | 1154056 | 1020487 | 1169183 | 1317090 | 1238383 | 1100602 | 1049261 | 916448 | 670409 | 529704 | 470858 | 363413 | 248541 | 139977 | 79927 |
| 2005 | Thyroid cancer | 0.00 | 0.00 | 0.00 | 0.17 | 0.52 | 0.69 | 1.03 | 1.37 | 1.86 | 2.54 | 2.86 | 2.95 | 3.43 | 3.21 | 4.46 | 1.93 | 5.63 | 2.86 | 0 |
| 2006 | Population | 120594 | 519974 | 784464 | 1085639 | 1042560 | 918813 | 1049050 | 1201671 | 1167421 | 1046486 | 974136 | 850732 | 604325 | 472689 | 417488 | 329336 | 225061 | 124208 | 74567 |
| 2006 | Thyroid cancer | 0.00 | 0.00 | 0.00 | 0.00 | 0.38 | 0.76 | 2 | 1.5 | 2.23 | 2.92 | 2.05 | 6.7 | 4.96 | 2.75 | 3.59 | 3.64 | 4 | 3.22 | 4.02 |
| 2007 | Population | 149716 | 610158 | 931680 | 1264384 | 1213378 | 1111889 | 1257645 | 1398319 | 1317286 | 1197710 | 1096739 | 963919 | 727851 | 583404 | 499443 | 379600 | 260467 | 150541 | 86044 |
| 2007 | Thyroid cancer | 0.00 | 0.00 | 0.00 | 0.00 | 0.91 | 0.54 | 0.95 | 1.57 | 2.05 | 3.76 | 4.29 | 4.98 | 3.57 | 3.43 | 3.8 | 4.74 | 4.22 | 4.65 | 2.32 |
| 2008 | Population | 114798 | 483284 | 723761 | 1053106 | 1013705 | 968502 | 1081644 | 1222148 | 1285827 | 1193285 | 1096844 | 986872 | 752264 | 598332 | 482261 | 394543 | 273871 | 158295 | 96947 |
| 2008 | Thyroid cancer | 0.00 | 0.00 | 0.00 | 0.00 | 0.39 | 0.93 | 1.39 | 1.96 | 3.5 | 4.19 | 4.47 | 6.79 | 5.45 | 5.52 | 5.6 | 3.55 | 4.38 | 3.16 | 5.16 |
| 2009 | Population | 235911 | 1041479 | 1502814 | 1916218 | 1900672 | 2151751 | 2226771 | 2360292 | 2452487 | 2390991 | 2258525 | 1872586 | 1618375 | 1239393 | 986179 | 770750 | 551789 | 317780 | 186750 |
| 2009 | Thyroid cancer | 0.00 | 0.1 | 0.07 | 0.16 | 0.63 | 0.98 | 2.11 | 2.41 | 4.65 | 3.43 | 4.96 | 6.3 | 5.93 | 6.29 | 5.78 | 3.89 | 4.35 | 2.52 | 2.14 |
| 2010 | Population | 444535 | 1845945 | 2436328 | 2772008 | 3066274 | 3599952 | 3274465 | 3434542 | 3852198 | 4049308 | 3637595 | 3027414 | 2720182 | 2038451 | 1525731 | 1222795 | 902954 | 519153 | 295106 |
| 2010 | Thyroid cancer | 0.00 | 0.00 | 0.04 | 0.25 | 0.72 | 1.11 | 1.83 | 2.45 | 2.93 | 4.17 | 6.02 | 5.55 | 5.7 | 5.25 | 4.59 | 4.33 | 3.1 | 1.73 | 1.02 |
| 2011 | Population | 608015 | 2530811 | 3116900 | 3364797 | 4000279 | 4992606 | 4444446 | 4366627 | 4907113 | 5191348 | 4874353 | 3789690 | 3620031 | 2657199 | 2019405 | 1541894 | 1157496 | 659694 | 376546 |
| 2011 | Thyroid cancer | 0.00 | 0.04 | 0.06 | 0.3 | 0.7 | 1.44 | 2.83 | 4.08 | 4.71 | 5.99 | 7.65 | 7.63 | 7.07 | 6.62 | 5.89 | 5.45 | 4.23 | 4.85 | 2.66 |
| 2012 | Population | 1034932 | 4246465 | 5109449 | 5403525 | 6547529 | 8303801 | 7478474 | 7191826 | 7893621 | 8902075 | 8282069 | 6287313 | 6326693 | 4731770 | 3437434 | 2621343 | 2001853 | 1154707 | 655418 |
| 2012 | Thyroid cancer | 0.00 | 0.00 | 0.1 | 0.15 | 0.76 | 1.78 | 2.7 | 4.38 | 5.7 | 7.43 | 9.36 | 8.62 | 8.68 | 7.27 | 6.46 | 5.42 | 3.9 | 3.12 | 2.9 |
| 2013 | Population | 1188267 | 5045688 | 6369010 | 6345890 | 7435288 | 9284188 | 8861382 | 8153750 | 8997873 | 10355298 | 9989740 | 7540579 | 7503803 | 5871692 | 4195278 | 3130066 | 2392336 | 1402448 | 836142 |
| 2013 | Thyroid cancer | 0.00 | 0.02 | 0.02 | 0.14 | 0.62 | 1.96 | 3.8 | 5.29 | 6.65 | 9.13 | 9.7 | 11.99 | 9.8 | 9.18 | 6.79 | 6.07 | 5.43 | 4.21 | 2.63 |
| 2014 | Population | 1538796 | 6469987 | 8044993 | 7742358 | 8857711 | 11274055 | 11590288 | 10106946 | 10922658 | 12635523 | 12619207 | 9899517 | 9246781 | 7731458 | 5482998 | 3966411 | 3039135 | 1863914 | 1148696 |
| 2014 | Thyroid cancer | 0.00 | 0.03 | 0.04 | 0.22 | 0.73 | 2.35 | 4.56 | 6.99 | 8.61 | 11.02 | 12.55 | 14.8 | 12.45 | 11.41 | 8.23 | 6.18 | 5.26 | 3.65 | 3.22 |
| 2015 | Population | 1667166 | 7691202 | 9515907 | 8725082 | 9582723 | 12241633 | 13802510 | 11700927 | 11982639 | 14116388 | 14990002 | 12207660 | 10238321 | 9372558 | 6697434 | 4893849 | 3576332 | 2260705 | 1527925 |
| 2015 | Thyroid cancer | 0.00 | 0.01 | 0.02 | 0.32 | 1.09 | 2.78 | 5.87 | 8.33 | 10.46 | 12.7 | 13.61 | 18.5 | 14.68 | 12.61 | 9.65 | 6.93 | 4.78 | 4.51 | 2.95 |
| 2016 | Population | 1964229 | 8740803 | 10980171 | 9977390 | 10991951 | 13455678 | 15125894 | 12713880 | 13733490 | 15636104 | 17024852 | 14101498 | 11412352 | 10908461 | 8010624 | 5628988 | 4107122 | 2663639 | 1759926 |
| 2016 | Thyroid cancer | 0.05 | 0.01 | 0.02 | 0.24 | 1.11 | 3.2 | 6.59 | 9.78 | 10.67 | 12.84 | 14.75 | 19.64 | 13.24 | 12.9 | 9.8 | 6.89 | 4.72 | 3.53 | 2.61 |
| 2017 | Population | 2400144 | 10380632 | 13110047 | 12121163 | 12967812 | 15574905 | 17111848 | 15294407 | 16360405 | 18003058 | 20114103 | 16567881 | 13426938 | 12863108 | 9745162 | 6885652 | 4931043 | 3172195 | 2060169 |
| 2017 | Thyroid cancer | 0.00 | 0.03 | 0.04 | 0.17 | 0.87 | 3.36 | 7.96 | 9.98 | 11.97 | 13.26 | 16.03 | 20.84 | 14.44 | 12.88 | 9.63 | 7.17 | 4.89 | 3.75 | 3.45 |
| 2018 | Population | 2943208 | 13384388 | 16771727 | 15694423 | 16368594 | 19856871 | 21427594 | 20195142 | 20793522 | 22569084 | 25457691 | 21589480 | 17906629 | 16512256 | 13084071 | 9093269 | 6441759 | 4196495 | 2826565 |
| 2018 | Thyroid cancer | 0.00 | 0.03 | 0.07 | 0.36 | 1.18 | 4 | 10.16 | 14.61 | 17.15 | 17.59 | 20.84 | 25.33 | 20.94 | 17.21 | 13.38 | 8.6 | 5.68 | 4.38 | 3.18 |

**S4 Table. Male coverage population and age-specific incidence rate of thyroid cancer in cancer registration areas of China, 2005-2018**

| Year | Male | 0- | 1-4 | 5-9 | 10-14 | 15-19 | 20-24 | 25-29 | 30-34 | 35-39 | 40-44 | 45-49 | 50-54 | 55-59 | 60-64 | 65-69 | 70-74 | 75-79 | 80-84 | 85+ |
| --- | --- | --- | --- | --- | --- | --- | --- | --- | --- | --- | --- | --- | --- | --- | --- | --- | --- | --- | --- | --- |
| 2005 | Population | 173406 | 795080 | 1315532 | 1717937 | 2195880 | 2468195 | 2135309 | 2297322 | 2399803 | 2563943 | 2461207 | 2061242 | 1431317 | 1033942 | 991763 | 853829 | 536139 | 264557 | 130846 |
| 2005 | Thyroid cancer | 0.00 | 0.00 | 0.00 | 0.06 | 0.27 | 0.89 | 1.03 | 1.31 | 1.83 | 3 | 2.72 | 3.35 | 3.7 | 4.35 | 3.83 | 3.75 | 5.04 | 4.91 | 3.06 |
| 2006 | Population | 192243 | 879471 | 1343415 | 1796678 | 2216555 | 2566537 | 2376828 | 2464449 | 2669541 | 2807802 | 2594751 | 2319331 | 1607211 | 1170392 | 1058385 | 930407 | 589749 | 289644 | 140329 |
| 2006 | Thyroid cancer | 0.00 | 0.00 | 0.00 | 0.11 | 0.41 | 1.09 | 1.68 | 1.87 | 2.02 | 2.42 | 2.66 | 4.35 | 3.48 | 3.16 | 4.16 | 5.59 | 5.93 | 4.49 | 3.56 |
| 2007 | Population | 214585 | 894983 | 1333850 | 1761150 | 2220957 | 2722091 | 2457773 | 2363922 | 2665650 | 2737200 | 2506456 | 2371821 | 1687358 | 1222016 | 1046599 | 939824 | 612209 | 314649 | 155744 |
| 2007 | Thyroid cancer | 0.00 | 0.00 | 0.22 | 0.06 | 0.36 | 0.88 | 1.99 | 2.07 | 2.78 | 3.07 | 3.67 | 4.38 | 3.67 | 2.86 | 4.78 | 5.11 | 4.74 | 4.77 | 3.21 |
| 2008 | Population | 230304 | 988089 | 1347405 | 1783907 | 2207619 | 2909458 | 2787889 | 2530927 | 2886770 | 2863495 | 2897277 | 2809713 | 2078657 | 1479943 | 1133807 | 1052543 | 752806 | 395524 | 204464 |
| 2008 | Thyroid cancer | 0.00 | 0.1 | 0.00 | 0.06 | 0.59 | 1.07 | 2.33 | 2.61 | 3.33 | 3.91 | 4.42 | 4.84 | 5.58 | 4.46 | 4.32 | 3.99 | 5.84 | 4.55 | 4.4 |
| 2009 | Population | 327151 | 1444017 | 1900139 | 2379996 | 2795480 | 3790457 | 3687009 | 3350468 | 3687707 | 3635160 | 3744392 | 3370296 | 2751754 | 1961417 | 1458084 | 1256564 | 933904 | 496334 | 261225 |
| 2009 | Thyroid cancer | 0.00 | 0.07 | 0.05 | 0.17 | 0.5 | 1.29 | 2.31 | 2.39 | 3.09 | 3.58 | 5.45 | 4.81 | 5.41 | 5.71 | 4.94 | 5.49 | 6.75 | 4.23 | 5.36 |
| 2010 | Population | 553502 | 2381653 | 3024624 | 3502563 | 3942503 | 5311134 | 5202735 | 4864799 | 5382560 | 5466769 | 5444970 | 4719319 | 4126860 | 2912878 | 2084787 | 1752290 | 1327608 | 709356 | 365311 |
| 2010 | Thyroid cancer | 0.00 | 0.00 | 0.00 | 0.09 | 0.41 | 1.19 | 2.36 | 2.96 | 3.34 | 3.29 | 4.28 | 5.23 | 5.5 | 5.39 | 4.17 | 3.54 | 3.92 | 2.96 | 3.01 |
| 2011 | Population | 684618 | 2936828 | 3538965 | 3921585 | 4580296 | 6037264 | 6017399 | 5711938 | 6111802 | 6410317 | 6400357 | 5282669 | 5024400 | 3571886 | 2534278 | 2021534 | 1565530 | 853731 | 437643 |
| 2011 | Thyroid cancer | 0.00 | 0.00 | 0.00 | 0.15 | 0.41 | 1.64 | 3.07 | 4.8 | 4.84 | 5.21 | 5.67 | 6.28 | 6.17 | 6.64 | 5.09 | 5.84 | 3.96 | 4.45 | 3.2 |
| 2012 | Population | 994252 | 4074266 | 4762697 | 5062836 | 6243621 | 8380644 | 8188498 | 7792141 | 8013956 | 8963360 | 8642568 | 6974532 | 7069983 | 5083547 | 3497777 | 2692482 | 2112988 | 1189503 | 631518 |
| 2012 | Thyroid cancer | 0.00 | 0.00 | 0.08 | 0.08 | 0.62 | 1.31 | 3.18 | 5.04 | 5.09 | 6.08 | 7.07 | 6.01 | 7.03 | 6.45 | 6.15 | 4.72 | 3.27 | 3.78 | 3.64 |
| 2013 | Population | 1120183 | 4793423 | 5806795 | 5683922 | 6949280 | 9187448 | 9218141 | 8709451 | 8914855 | 10196645 | 9886294 | 8125627 | 8211309 | 6143354 | 4176473 | 3104989 | 2441299 | 1414709 | 776142 |
| 2013 | Thyroid cancer | 0.00 | 0.04 | 0.03 | 0.11 | 0.39 | 1.63 | 3.97 | 6.56 | 6.73 | 7.55 | 7.17 | 8.33 | 8.1 | 7.55 | 5.91 | 4.77 | 4.3 | 4.24 | 2.83 |
| 2014 | Population | 1492182 | 6311799 | 7508364 | 7183868 | 8504790 | 11413925 | 11962976 | 10979497 | 11195114 | 12704250 | 12564439 | 10614514 | 10017016 | 8078525 | 5558957 | 3999592 | 3121472 | 1901893 | 1090718 |
| 2014 | Thyroid cancer | 0.00 | 0.05 | 0.04 | 0.18 | 0.44 | 1.73 | 5.23 | 8.26 | 8.85 | 9.44 | 8.86 | 11.09 | 9.88 | 8.5 | 7.32 | 5.7 | 5.13 | 3.68 | 3.39 |
| 2015 | Population | 1603833 | 7337612 | 8628305 | 7984556 | 9049253 | 11875899 | 13463823 | 12246457 | 12103610 | 13662267 | 14203282 | 12370248 | 10663349 | 9374709 | 6514220 | 4665784 | 3483593 | 2197248 | 1334999 |
| 2015 | Thyroid cancer | 0.00 | 0.00 | 0.02 | 0.15 | 0.66 | 2.1 | 5.72 | 9.04 | 9.91 | 9.36 | 8.77 | 11.07 | 9.89 | 8.83 | 6.55 | 5.96 | 4.36 | 4.28 | 3.82 |
| 2016 | Population | 2027078 | 8826047 | 10617166 | 9693183 | 10812109 | 13604438 | 15434056 | 14042473 | 14671949 | 15943173 | 17236389 | 14678137 | 12352675 | 11431650 | 8173167 | 5655313 | 4133505 | 2657823 | 1621991 |
| 2016 | Thyroid cancer | 0.1 | 0.00 | 0.05 | 0.1 | 0.48 | 2.12 | 6.27 | 9.98 | 9.6 | 9.66 | 8.96 | 11.08 | 9.01 | 9.29 | 7.11 | 5.5 | 4.55 | 3.65 | 2.53 |
| 2017 | Population | 2465356 | 10544615 | 12744732 | 11546149 | 12216721 | 14815642 | 16839085 | 16005637 | 16837620 | 17691143 | 19764144 | 16694834 | 13778674 | 13250102 | 9698625 | 6654237 | 4725730 | 3027487 | 1834427 |
| 2017 | Thyroid cancer | 0.00 | 0.00 | 0.04 | 0.09 | 0.65 | 2.52 | 6.85 | 10.4 | 10.99 | 9.98 | 9.73 | 11.59 | 8.95 | 8.63 | 7.07 | 5.65 | 4.51 | 3.44 | 3.11 |
| 2018 | Population | 2831943 | 12729464 | 15279403 | 14201472 | 14585468 | 17702871 | 19615180 | 19464756 | 20154570 | 20862589 | 23439973 | 19869722 | 16963128 | 15772987 | 12153174 | 8276466 | 5682100 | 3644185 | 2259098 |
| 2018 | Thyroid cancer | 0.00 | 0.02 | 0.02 | 0.17 | 0.56 | 2.92 | 8.78 | 12.67 | 13.94 | 12.17 | 11.54 | 12.98 | 11.55 | 11.01 | 8.52 | 6.46 | 4.4 | 4.09 | 3.54 |

**S5 Table. Female coverage population and age-specific incidence rate of thyroid cancer in cancer registration areas of China, 2005-2018**

| Year | Female | 0- | 1-4 | 5-9 | 10-14 | 15-19 | 20-24 | 25-29 | 30-34 | 35-39 | 40-44 | 45-49 | 50-54 | 55-59 | 60-64 | 65-69 | 70-74 | 75-79 | 80-84 | 85+ |
| --- | --- | --- | --- | --- | --- | --- | --- | --- | --- | --- | --- | --- | --- | --- | --- | --- | --- | --- | --- | --- |
| 2005 | Population | 155305 | 704192 | 1181344 | 1590127 | 2066136 | 2268440 | 2050701 | 2269902 | 2258323 | 2444537 | 2381166 | 2032322 | 1409502 | 1075480 | 1057865 | 927751 | 628733 | 363891 | 230721 |
| 2005 | Thyroid cancer | 0.00 | 0.00 | 0.17 | 0.5 | 1.21 | 3.26 | 5.02 | 5.33 | 6.95 | 10.68 | 11.34 | 13.04 | 10.78 | 10.04 | 9.74 | 8.41 | 8.11 | 5.5 | 5.63 |
| 2006 | Population | 174563 | 785464 | 1211038 | 1667036 | 2129653 | 2420224 | 2312567 | 2449914 | 2642616 | 2683690 | 2503272 | 2285926 | 1588109 | 1198855 | 1125686 | 1021043 | 696740 | 403278 | 253930 |
| 2006 | Thyroid cancer | 0.00 | 0.00 | 0.25 | 0.48 | 1.13 | 3.51 | 6.14 | 6.98 | 9.23 | 11.22 | 12.98 | 16.54 | 13.92 | 10.01 | 8.79 | 10.97 | 5.31 | 8.68 | 5.12 |
| 2007 | Population | 191701 | 797041 | 1200021 | 1619203 | 2109163 | 2519749 | 2361136 | 2337215 | 2636017 | 2628619 | 2414244 | 2323058 | 1665629 | 1250034 | 1111722 | 1028102 | 704616 | 417135 | 266072 |
| 2007 | Thyroid cancer | 0.00 | 0.13 | 0.25 | 0.25 | 2.13 | 3.33 | 5.63 | 8.13 | 8.35 | 12.06 | 14.08 | 18.73 | 13.87 | 11.92 | 10.97 | 9.82 | 7.66 | 8.63 | 5.64 |
| 2008 | Population | 204918 | 885202 | 1215489 | 1645564 | 2096264 | 2723334 | 2684460 | 2498796 | 2870390 | 2788576 | 2793057 | 2739322 | 2066242 | 1503302 | 1200246 | 1160933 | 864151 | 516355 | 341586 |
| 2008 | Thyroid cancer | 0.00 | 0.00 | 0.33 | 0.24 | 1.81 | 4.19 | 7.79 | 8.84 | 11.64 | 13.48 | 18.62 | 21.57 | 18.25 | 13.97 | 12.66 | 12.06 | 8.33 | 7.55 | 12 |
| 2009 | Population | 292891 | 1289140 | 1711885 | 2178671 | 2631293 | 3564942 | 3553884 | 3294092 | 3643974 | 3561545 | 3609939 | 3268616 | 2733907 | 1954947 | 1492917 | 1367135 | 1051034 | 625393 | 412763 |
| 2009 | Thyroid cancer | 0.00 | 0.08 | 0.18 | 0.6 | 1.9 | 4.01 | 7.91 | 9.26 | 11.8 | 12.13 | 17.23 | 20.44 | 20.08 | 15.19 | 11.39 | 10.97 | 8.28 | 7.04 | 4.36 |
| 2010 | Population | 486435 | 2096825 | 2667249 | 3129573 | 3689182 | 5091617 | 5055499 | 4809513 | 5313020 | 5371195 | 5255654 | 4552651 | 4084984 | 2887210 | 2141523 | 1908171 | 1529005 | 917595 | 589813 |
| 2010 | Thyroid cancer | 0.00 | 0.00 | 0.11 | 0.54 | 1.49 | 3.69 | 7.26 | 9.67 | 10.8 | 12.49 | 17.24 | 19.77 | 18.7 | 14.06 | 11.39 | 8.75 | 8.04 | 5.99 | 4.92 |
| 2011 | Population | 612356 | 2589186 | 3152099 | 3501968 | 4242645 | 5838711 | 5908775 | 5633034 | 6070466 | 6349482 | 6228983 | 5084474 | 5008514 | 3542509 | 2579211 | 2169921 | 1803533 | 1086017 | 703547 |
| 2011 | Thyroid cancer | 0.00 | 0.04 | 0.16 | 0.31 | 1.6 | 4.52 | 9.16 | 12.25 | 14.08 | 16.49 | 22.03 | 23.4 | 21.54 | 18.38 | 14.35 | 10.65 | 6.93 | 7.09 | 5.4 |
| 2012 | Population | 882447 | 3564903 | 4204192 | 4422732 | 5705686 | 8044047 | 7960860 | 7627051 | 7913141 | 8866584 | 8449493 | 6699404 | 6985878 | 5028979 | 3543202 | 2870922 | 2428431 | 1507561 | 983724 |
| 2012 | Thyroid cancer | 0.00 | 0.00 | 0.07 | 0.41 | 1.86 | 5.08 | 9.76 | 13.86 | 16.1 | 20.09 | 24.06 | 26.42 | 23.91 | 19.9 | 15.21 | 10.76 | 8.19 | 5.24 | 4.07 |
| 2013 | Population | 991430 | 4158047 | 5132697 | 4960433 | 6292296 | 8800228 | 9007691 | 8515951 | 8773968 | 10066117 | 9706885 | 7816449 | 8061197 | 6086285 | 4237380 | 3291473 | 2785738 | 1762244 | 1187642 |
| 2013 | Thyroid cancer | 0.00 | 0.02 | 0.06 | 0.38 | 1.72 | 4.92 | 11.07 | 17.27 | 19.41 | 22.77 | 26.31 | 32.5 | 28.28 | 24.02 | 16.33 | 12.37 | 10.34 | 5.84 | 5.64 |
| 2014 | Population | 1321862 | 5478844 | 6650453 | 6293607 | 7774163 | 10856412 | 11691822 | 10798011 | 10983789 | 12450713 | 12307504 | 10246073 | 9861510 | 8062979 | 5626648 | 4198659 | 3502144 | 2318947 | 1615316 |
| 2014 | Thyroid cancer | 0.08 | 0.00 | 0.09 | 0.37 | 1.87 | 5.82 | 12.78 | 20.56 | 23.95 | 28.45 | 30.45 | 40.52 | 35.22 | 28.98 | 19.98 | 12.77 | 8.77 | 6.64 | 3.96 |
| 2015 | Population | 1416150 | 6386000 | 7640855 | 6915197 | 8205951 | 11296870 | 13182611 | 12071550 | 11876947 | 13406894 | 13962710 | 11997873 | 10455327 | 9366577 | 6588261 | 4866170 | 3875659 | 2670297 | 1970903 |
| 2015 | Thyroid cancer | 0.00 | 0.02 | 0.07 | 0.55 | 2.19 | 6.69 | 15.89 | 23.68 | 26.4 | 30.51 | 31.46 | 43.85 | 37.13 | 29.85 | 20.72 | 13.13 | 8.31 | 5.99 | 4.72 |
| 2016 | Population | 1788641 | 7681746 | 9410149 | 8453698 | 9821121 | 12975979 | 15057787 | 13846099 | 14393216 | 15571517 | 16937078 | 14201829 | 12131389 | 11386814 | 8235724 | 5882212 | 4579432 | 3214901 | 2363767 |
| 2016 | Thyroid cancer | 0.06 | 0.05 | 0.04 | 0.44 | 2.15 | 7.11 | 16.24 | 24.96 | 26.48 | 29.77 | 32.7 | 45.85 | 33.58 | 29.54 | 20.9 | 12.95 | 8.19 | 5.91 | 4.99 |
| 2017 | Population | 2188292 | 9190787 | 11290100 | 10042339 | 11049673 | 14199254 | 16494026 | 15952169 | 16658626 | 17374265 | 19536061 | 16196844 | 13539831 | 13144245 | 9780861 | 6881776 | 5238141 | 3699088 | 2745617 |
| 2017 | Thyroid cancer | 0.00 | 0.03 | 0.13 | 0.33 | 1.79 | 7.67 | 19.03 | 26.18 | 29.64 | 31.03 | 35.36 | 47.06 | 34.7 | 30.95 | 21.44 | 13.43 | 8.69 | 6.65 | 4.48 |
| 2018 | Population | 2519946 | 11201774 | 13562972 | 12390011 | 13142326 | 16812239 | 18990123 | 19198385 | 19801472 | 20383467 | 23040048 | 19306199 | 16708862 | 15611141 | 12319000 | 8547333 | 6302079 | 4456919 | 3377404 |
| 2018 | Thyroid cancer | 0.00 | 0.04 | 0.1 | 0.56 | 2.26 | 7.83 | 21.15 | 31.51 | 35.73 | 36.78 | 41.9 | 49.61 | 42.14 | 35.14 | 24.67 | 14.3 | 9.06 | 6.64 | 4.94 |
