# Supplementary material for: Spatial and temporal distribution of thyroid cancer incidence in China: An ecological study based on a national registry
Source: PLoS One. 2026 Mar 20;21(3):e0341734. doi: 10.1371/journal.pone.0341734 (PMC13004386; doi:10.1371/journal.pone.0341734)
Supplement: Attachment — Submitted filename: PONE-D-25-46157_1st reviewer- Comments.pdf [file pone.0341734.s002.pdf]

PLOS ONE

Spatial and temporal distribution of thyroid cancer incidence in China: A study based on a national registry

--Manuscript Draft--

**Manuscript Number:**	PONE-D-25-46157
**Article Type:**	Research Article
**Full Title:**	Spatial and temporal distribution of thyroid cancer incidence in China: A study based on a national registry
**Short Title:**	Spatial and temporal distribution of thyroid cancer incidence in China
**Corresponding Author:**	Hao Liu<br>First Affiliated Hospital of Shihezi University School of Medicine<br>Shihezi, Xinjiang CHINA
**Keywords:**	Cancer registry; Thyroid cancer; Incidence; Spatial autocorrelation
**Abstract:**	The objective of this study was to analyze the incidence rate of thyroid cancer in China to elucidate its spatiotemporal distribution and to provide an evidence-based foundation for prevention and control strategies. Thyroid cancer incidence data from 2005 to 2018 were obtained from the China Cancer Registry Annual Reports (2008–2021). The Joinpoint regression model was used to describe the time trend, the age-period-cohort model was employed to analyze the effects of age, period and cohort on the thyroid cancer incidence, and spatial autocorrelation was used to analyze its spatial distribution. Results showed China's age-standardized incidence rate (ASIR) of thyroid cancer from 2005 to 2018 was 8.69 per 100,000, exhibiting an upward trend with an average annual percent change (AAPC) of 10.7% (95% CI: 9.5%, 11.9%). The ASIR was higher in urban than in rural areas, though its growth rate was lower than in rural areas; similarly, it was higher in females than males but grew slower than in males. Nationally, incidence risk initially increased and then declined with advancing age, while period effects showed an upward trend and cohort effects a downward trend. Spatial autocorrelation revealed clustered incidence patterns, with northeastern China exhibiting a high-high clustering. The national thyroid cancer surveillance system should be strengthened, a registration system for high-risk populations should be established, and greater investment should be allocated to etiological research. The scientific nature of prevention and control efforts should be improved, and the risk of diseases can be reduced through measures such as controlling weight and regulating radiation. These measures when coordinated, can contribute to preventing thyroid cancer.
**Order of Authors:**	Wen Liu
	Jiaqi Yang
	Zhenhao Zheng
	Yang Yu
	Jun Li
	Ting Ma
	Hao Liu
**Additional Information:**	
**Question**	**Response**
**Financial Disclosure**<br><br>Enter a financial disclosure statement that describes the sources of funding for the work included in this submission. Review the submission guidelines for detailed	Yes

Powered by Editorial Manager® and ProduXion Manager® from Aries Systems Corporation

requirements. View published research articles from *PLOS ONE* for specific examples.<br><br>This statement is required for submission and **will appear in the published article** if the submission is accepted. Please make sure it is accurate.<br><br>**Funded studies**<br>Enter a statement with the following details:<br>• Initials of the authors who received each award<br>• Grant numbers awarded to each author<br>• The full name of each funder<br>• URL of each funder website<br>• Did the sponsors or funders play any role in the study design, data collection and analysis, decision to publish, or preparation of the manuscript?<br><br>Did you receive funding for this work?	
Please add funding details.<br>as follow-up to "**Financial Disclosure**<br><br>Enter a financial disclosure statement that describes the sources of funding for the work included in this submission. Review the submission guidelines for detailed requirements. View published research articles from *PLOS ONE* for specific examples.<br><br>This statement is required for submission and **will appear in the published article** if the submission is accepted. Please make sure it is accurate.	This work was supported by Bingtuan Science and Technology Programs (2023ZD004,2023ZD009).

**Funded studies**<br>Enter a statement with the following details:<br>• Initials of the authors who received each award<br>• Grant numbers awarded to each author<br>• The full name of each funder<br>• URL of each funder website<br>• Did the sponsors or funders play any role in the study design, data collection and analysis, decision to publish, or preparation of the manuscript?<br><br>Did you receive funding for this work?"	
Please select the country of your main research funder (please select carefully as in some cases this is used in fee calculation).<br>as follow-up to "**Financial Disclosure**<br><br>Enter a financial disclosure statement that describes the sources of funding for the work included in this submission. Review the submission guidelines for detailed requirements. View published research articles from *PLOS ONE* for specific examples.<br><br>This statement is required for submission and **will appear in the published article** if the submission is accepted. Please make sure it is accurate.<br><br>**Funded studies**<br>Enter a statement with the following details:<br>• Initials of the authors who received each award<br>• Grant numbers awarded to each author<br>• The full name of each funder<br>• URL of each funder website<br>• Did the sponsors or funders play any role in the study design, data collection and analysis, decision to publish, or preparation of the manuscript?<br><br>Did you receive funding for this work?"	CHINA - CN
**Competing Interests**	The authors have declared that no competing interests exist.

Use the instructions below to enter a competing interest statement for this submission. On behalf of all authors, disclose any competing interests that could be perceived to bias this work—acknowledging all financial support and any other relevant financial or non-financial competing interests.<br><br>This statement is required for submission and **will appear in the published article** if the submission is accepted. Please make sure it is accurate and that any funding sources listed in your Funding Information later in the submission form are also declared in your Financial Disclosure statement.<br><br>View published research articles from *PLOS ONE* for specific examples.<br><br>**NO authors have competing interests**<br><br>Enter: *The authors have declared that no competing interests exist.*<br><br>**Authors with competing interests**<br><br>Enter competing interest details beginning with this statement:<br><br>*I have read the journal's policy and the authors of this manuscript have the following competing interests: [insert competing interests here]*<br><br>* typeset	
**Ethics Statement**<br><br>Enter an ethics statement for this submission. This statement is required if the study involved:<br><br>• Human participants<br>• Human specimens or tissue<br>• Vertebrate animals or cephalopods<br>• Vertebrate embryos or tissues	N/A

Powered by Editorial Manager® and ProduXion Manager® from Aries Systems Corporation

• Field research<br><br>Write "N/A" if the submission does not require an ethics statement.<br><br>General guidance is provided below. Consult the submission guidelines for detailed instructions. **Make sure that all information entered here is included in the Methods section of the manuscript.**	

Powered by Editorial Manager® and ProduXion Manager® from Aries Systems Corporation

**Format for specific study types**<br><br>**Human Subject Research (involving human participants and/or tissue)**<br>• Give the name of the institutional review board or ethics committee that approved the study<br>• Include the approval number and/or a statement indicating approval of this research<br>• Indicate the form of consent obtained (written/oral) or the reason that consent was not obtained (e.g. the data were analyzed anonymously)<br><br>**Animal Research (involving vertebrate animals, embryos or tissues)**<br>• Provide the name of the Institutional Animal Care and Use Committee (IACUC) or other relevant ethics board that reviewed the study protocol, and indicate whether they approved this research or granted a formal waiver of ethical approval<br>• Include an approval number if one was obtained<br>• If the study involved *non-human primates*, add *additional details* about animal welfare and steps taken to ameliorate suffering<br>• If anesthesia, euthanasia, or any kind of animal sacrifice is part of the study, include briefly which substances and/or methods were applied<br><br>**Field Research**<br><br>Include the following details if this study involves the collection of plant, animal, or other materials from a natural setting:<br>• Field permit number<br>• Name of the institution or relevant body that granted permission	
**Data Availability**<br><br>Authors are required to make all data underlying the findings described fully available, without restriction, and from the time of publication. PLOS allows rare exceptions to address legal and ethical concerns. See the PLOS Data Policy and FAQ for detailed information.	Yes - all data are fully available without restriction

A Data Availability Statement describing where the data can be found is required at submission. Your answers to this question constitute the Data Availability Statement and **will be published in the article**, if accepted.<br><br>**Important:** Stating 'data available on request from the author' is not sufficient. If your data are only available upon request, select 'No' for the first question and explain your exceptional situation in the text box.<br><br>Do the authors confirm that all data underlying the findings described in their manuscript are fully available without restriction?	
**Describe where the data may be found in full sentences. If you are copying our sample text, replace any instances of XXX with the appropriate details.**<br><br>• If the data are **held or will be held in a public repository**, include URLs, accession numbers or DOIs. If this information will only be available after acceptance, indicate this by ticking the box below. For example: *All XXX files are available from the XXX database (accession number(s) XXX, XXX.).*<br>• If the data are all contained **within the manuscript and/or Supporting Information files**, enter the following: *All relevant data are within the manuscript and its Supporting Information files.*<br>• If neither of these applies but you are able to provide **details of access elsewhere**, with or without limitations, please do so. For example:<br><br>*Data cannot be shared publicly because of [XXX]. Data are available from the XXX Institutional Data Access / Ethics Committee (contact via XXX) for researchers who meet the criteria for access to confidential data.*<br><br>*The data underlying the results presented in the study are available from (include the name of the third party*	The data is sourced from the China Cancer Registry Annual Report (2008-2021) released by the National Cancer Center of China.

and contact information or URL).<br>• This text is appropriate if the data are owned by a third party and authors do not have permission to share the data.<br><br>* typeset	
Additional data availability information:	

Manuscript Click here to access/download;Manuscript;manuscript .docx

Title page

Title: Spatial and temporal distribution of thyroid cancer incidence in China: A study based on a national registry

Author's information:

Names: Wen Liu[1] *, Jiaqi Yang[2] *, Zhenhao Zheng[1], Yang Yu[1], Jun Li[1, †], Ting Ma[1, †], Hao Liu[3, †]

* Contributed equally to this work.

† Correspondence: 1287424798@qq.com (JL), 83661825@qq.com (TM), liuhao850619@sina.com (HL)

Institutional addresses:

[1] Department of Ultrasound, the First Affiliated Hospital of Shihezi University, Shihezi, Xinjiang, China.

[2] Department of Preventive Medicine, School of Medicine, Shihezi University, Shihezi, Xinjiang, China

[3] Department of Gastroenterology, the First Affiliated Hospital of Shihezi University, Shihezi, Xinjiang, China

Emails: 13779205189@163.com (Wen Liu), 19891023083@163.com (Jiaqi Yang), 1181656974@qq.com (Zhenhao Zheng), 1084825085@qq.com (Yang Yu), 1287424798@qq.com (Jun Li), 83661825@qq.com (Ting Ma), liuhao850619@sina.com (Hao Liu)

Correspondent authors:

1287424798@qq.com (JL)

83661825@qq.com (TM)

liuhao850619@sina.com (HL)

Department of Ultrasound, the First Affiliated Hospital of Shihezi University, Shihezi, Xingjiang, China

Department of Gastroenterology, the First Affiliated Hospital of Shihezi University, Shihezi, Xinjiang, China

Spatial and temporal distribution of thyroid cancer incidence in China: A study based on a national registry

Abstract

The objective of this study was to analyze the incidence rate of thyroid cancer in China to elucidate its spatiotemporal distribution and to provide an evidence-based foundation for prevention and control strategies. Thyroid cancer incidence data from 2005 to 2018 were obtained from the China Cancer Registry Annual Reports (2008–2021). The Joinpoint regression model was used to describe the time trend, the age-period-cohort model was employed to analyze the effects of age, period and cohort on the thyroid cancer incidence, and spatial autocorrelation was used to analyze its spatial distribution. Results showed China's age-standardized incidence rate (ASIR) of thyroid cancer from 2005 to 2018 was 8.69 per 100,000, exhibiting an upward trend with an average annual percent change (AAPC) of 10.7% (95% CI: 9.5%, 11.9%). The ASIR was higher in urban than in rural areas, though its growth rate was lower than in rural areas; similarly, it was higher in females than males but grew slower than in males. Nationally, incidence risk initially increased and then declined with advancing age, while period effects showed an upward trend and cohort effects a downward trend. Spatial autocorrelation revealed clustered incidence patterns, with northeastern China exhibiting a high-high clustering. The national thyroid cancer surveillance system should be strengthened, a registration system for high-risk populations should be established, and greater investment should be

allocated to etiological research. The scientific nature of prevention and control efforts should be improved, and the risk of diseases can be reduced through measures such as controlling weight and regulating radiation. These measures when coordinated, can contribute to preventing thyroid cancer.

Introduction

Thyroid cancer is a common malignancy of the endocrine system. Over the past few decades, the global incidence rate of thyroid cancer has exhibited a rapid upward trend [1]. According to data from the International Agency for Research on Cancer (IARC), there were approximately 821,000 new cases of thyroid cancer worldwide in 2022, an increase of about 235,000 compared with 2020 (586,000 cases), making it the seventh most common cancer globally [1, 2]. In China, the burden of thyroid cancer is comparably severe. In 2022, the number of new thyroid cancer cases in China reached 466,000, accounting for 9.7% of all new cancer cases in the country, and its incidence rate rank rose to the third place [3], posing a significant threat to the health of Chinese residents.

In recent years, with advancements in diagnostic and therapeutic technologies, the survival of patients with thyroid cancer has been markedly extended. However, the consequent long-term treatment demands, frequent follow-up requirements, and ongoing medical care have imposed unprecedented pressure on healthcare resources [4, 5]. Data indicate that disability-adjusted life years (DALYs) for thyroid cancer in China increased from 1990 to 2019 [6], reflecting a continuously escalating health burden on the population. In light of this situation, it is particularly important to strengthen prevention systems, optimize early screening programs, and refine comprehensive management strategies [7].

Comprehensive understanding of the epidemic characteristics of diseases is a key prerequisite for establishing an effective prevention and control system. However, at

present, there are relatively few studies that comprehensively describe the temporal and spatial epidemic characteristics of thyroid cancer across the entire country of China from multiple perspectives. Based on 14–years of data from the China Cancer Registry Annual Report from 2008 to 2021, this study analyzed the epidemic characteristics of thyroid cancer incidence rate and its age-period-cohort effects, and used spatial autocorrelation methods to analyze the spatial distribution characteristics. These analysis will facilitate understanding of the temporal and spatial distribution and epidemiological trends of thyroid cancer in China, provide a foundation for formulating targeted prevention strategies, and offer a reference for other countries around the world.

Methods

Data Sources

The 2005–2018 thyroid cancer incidence rate data in this study were obtained from the reported data of 947 cancer registries across 31 provinces (autonomous regions, municipalities directly under the Central Government) and Xinjiang Production and Construction Corps (excluding the Hong Kong Special Administrative Region, Macao Special Administrative Region, and Taiwan Province) in the China Cancer Registry Annual Report from 2008 to 2021 [8-21]. After data verification and quality control, data from 700 cancer registries were included. The data mainly covered the incidence and mortality rate data of all combined cancers and 22 types of cancers, with comparisons of cancer distribution differences by region, age, and gender. By the end of 2022, cancer registration had covered 99.8% of the national population. Cancer

registration regions formulated work specifications and quality control procedures at various stages, ensuring comparability, timeliness, completeness, and validity.

Classification Standards

The urban-rural classification was based on the Codes for the administrative divisions of the People's Republic of China (GB/T2260-2007), with cities at or above the prefecture level classified as urban areas and counties and county-level cities classified as rural areas, while also considering factors such as regional economy and lifestyle. The diagnostic criteria for thyroid cancer was based on the International Classification of Diseases, 10th Revision (ICD-10/C73) [22].

Statistical Indicators

Incidence rate, also known as crude incidence rate, refers to the number of new cancer cases registered per 100,000 population in a given year, and reflects the incidence level of the population. Since the crude incidence rate is significantly affected by the age composition of the population, when comparing and analyzing the incidence rates in different regions or the incidence levels of the same population group in different periods, it is necessary to calculate the age-standardized incidence rate (ASIR) to eliminate the impact of population age structure on the incidence level. The ASIR refers to the incidence rate calculated according to the age structure of a certain standard population. The standard population data in this study were obtained from the Sixth National Population Census published by the National Bureau of Statistics of China in 2010 [23].

$$\text{Incidence rate per 100,000}=\frac{\text{new cases occurring during a given time period}}{\text{population at risk during the same time period}}\times 100{,}000$$

$$\text{ASIR}=\frac{\sum \text{standard population in corresponding age group} \times \text{age-specific incidence rate}}{\sum \text{standard population}}\times 100{,}000$$

Joinpoint regression model

The Joinpoint regression model is a statistical method used to analyze the continuous temporal trends of disease incidence rates, and evaluate the trend change characteristics of different segments within the global time range [24]. By setting multiple joinpoints, the study period is divided into different intervals, and trend fitting and optimization are performed separately within each interval. This enables a more detailed assessment of the specific change characteristics of diseases in different intervals over the entire time range. The Joinpoint regression model has two forms: the linear model (y = xb) and the log-linear model (lny = xb). Generally, the log-linear model is selected to analyze population-based cancer incidence rate trends. The Monte Carlo permutation test is used to determine the number of joinpoints, the location of each joinpoint, and the corresponding *P*-values, with the significance level set at $\alpha = 0.05$. By identifying the turning points, the long-term trend of disease incidence rate is divided into several statistically significant intervals [24-26]. The log-linear regression equation is as follows:

$$E[y/x]=e^{\beta_0+\beta_1+\delta_1(x-\tau_1)^{+}+\cdots+\delta_K(x-\tau_K)^{+}}$$

Where, e is the base of the natural logarithm, k is the number of turning points, τ_k is the number of unknown turning points, β_0 is the invariant parameter, β_1 is the regression coefficient, $\delta_k$indicates the slope of the k-th segmented function. When $(x-\tau_k)>0$, $(x-\tau_1)^{+}=x-\tau_k$; otherwise, $(x-\tau_1)^{+}=0$.

The incidence rate trend and its statistical significance were described by

calculating the annual percentage change (APC), average annual percentage change (AAPC), and their 95% confidence intervals (95% CI). The APC was used to describe the incidence rate trend in a certain time period, while the AAPC was used to comprehensively evaluate the overall average change trend of the incidence rate, focusing on the overall trend over time and eliminating short-term fluctuations, so it is more instructive in evaluating long-term trends.

$$APC=\left(e^{\beta_1}-1\right)\times 100$$

$$AAPC=\left(e^{\Sigma \omega_i \beta_i / \Sigma \omega_i}-1\right)\times 100$$

Where, β_1 is the regression coefficient, β_i is the regression coefficient corresponding to each interval, ω_i is the width of the interval span of each piece wise function. When the number of inflection points is 0, APC = AAPC. If APC > 0 or AAPC > 0 and 95% CI > 0, it indicates an upward trend; if APC < 0 or AAPC < 0 and 95% CI < 0, it indicates a downward trend. A value equal to 0 indicates no obvious trend.

Age-period-cohort model

The construction of the age-period-cohort model was based on the Poisson distribution [27]. After controlling for the interactive effects of variables such as age, period, and birth cohort, it systematically analyzed the influence of these three factors on disease incidence rate through the construction of statistical models and parameter estimation methods. Due to the high linear relationship between age, period, and cohort, neither the least squares method nor the maximum likelihood estimation method can obtain a unique estimate of the equation. To solve this problem, scholars

have proposed methods such as the Penalized Function Approach, Intrinsic Estimator (IE), Estimating Functions Approach, and Localized Constraints Method. This study adopted the age-period-cohort model combined with the IE to address the issue of multicollinearity and analyzed the impact of age, period, and cohort on the incidence rate of thyroid cancer. This method has been used in a number of epidemiological studies [28-30].

$$Y=\log(M)=\mu+\alpha age_1+\beta period_1+\gamma cohort_1+\varepsilon$$

Where, M represents the incidence rate of age groups, α represents the age effect, β represents the period effect, γ represents the cohort effect, μ is the intercept; ε is the random error.

In this study, age groups were divided at 5-year intervals, with three period groups (2005, 2010, 2015) and 19 cohort groups (1920–1924, 1925–1929, ..., 2015–2019).

Spatial autocorrelation analysis

Spatial autocorrelation can be used to evaluate whether interdependence exists between the values of a variable within the study area. Several indices are available to measure the degree of spatial autocorrelation, among which Moran's *I*, Geary's C, and G-statistics are commonly used. In this study, Moran's *I* statistic was employed to perform both global and local spatial autocorrelation analyses[31].

Global spatial autocorrelation is used to examine the spatial distribution characteristics of thyroid cancer incidence rate across the entire study area and to determine whether clustering occurs at an overall level. The global Moran's *I* index

ranges from -1 to 1. A value greater than 0 and close to 1 indicates strong positive spatial autocorrelation, suggesting a clustered pattern. Conversely, a value less than 0 and close to -1 indicates dispersion.

Local spatial autocorrelation examines the correlation between a specific spatial unit and its surrounding units. The local Moran's *I* index was used to identify four types of local spatial clustering patterns: high-high cluster, low-low cluster, high-low outlier, and low-high outlier.

Since provincial cancer data in China were only reported in the China Cancer Registry Annual Report from 2013 to 2019, this study applied spatial autocorrelation analysis to investigate the spatial distribution characteristics of thyroid cancer incidence rate between 2010 and 2016.

Statistical Methods

Joinpoint regression model was performed using Joinpoint Regression Program 5.0.2 software. The age-period-cohort model was analyzed using STATA 18. Spatial autocorrelation analysis was performed using Arc GIS 10.8 software. The test level was set at $\alpha=0.05$.

Results

Incidence rate and its trends of thyroid cancer in China

From 2005 to 2018, there were a total of 351,330 cases of thyroid cancer in China, of which 240,788 (68.54%) were in urban areas and 110,542 (31.46%) were in rural areas, with 85,622 (24.37%) in males and 265,708 (75.63%) in females. The incidence rate of thyroid cancer in China was 11.82 per 100,000, of which 15.42 per

100,000 were urban areas, 7.84 per 100,000 were rural areas, 5.69 per 100,000 were males, and 18.13 per 100,000 were females. The ASIR was 8.69 per 100,000, of which 11.40 per 100,000 were urban areas, 5.08 per 100,000 were rural areas, 4.18 per 100,000 were males, and 13.28 per 100,000 were females.

Between 2005 and 2018, the ASIR increased from 4.11/100,000 to 15.39/100,000, with an AAPC of 10.7% (95% CI: 9.5%, 11.9%). The AAPC for urban areas was 11.0% (95% CI: 11.0%, 13.8%), while for rural areas it was 15.2% (95% CI: 13.7%, 16.8%). For males, the AAPC was 11.5% (95% CI: 10.2%, 12.8%), and for females, it was 10.5% (95% CI: 9.3%, 11.7%). (Table 1, Table 2, Fig 1)

Table 1 Incidence rate of thyroid cancer in China, 2005-2018 (1/100,000)

Year	National			Urban			Rural			Male			Female		
	Case	Rate	ASIR	Case	Rate	ASIR	Case	Rate	ASIR	Case	Rate	ASIR	Case	Rate	ASIR
2005	2361	4.3	4.11	2122	5.22	4.84	239	1.68	1.76	550	1.98	1.92	1811	6.68	6.33
2006	2977	5.00	4.69	2697	5.79	5.31	280	2.15	2.14	659	2.2	2.07	2318	7.84	7.35
2007	3212	5.37	5.04	2891	6.48	5.90	321	2.11	2.21	732	2.42	2.29	2480	8.38	7.83
2008	4435	6.71	6.14	4035	7.74	6.98	400	2.86	2.80	993	2.98	2.76	3442	10.49	9.56
2009	5607	6.56	6.11	4742	8.25	7.51	865	3.09	3.02	1344	3.11	2.91	4263	10.09	9.36
2010	7740	6.21	5.82	6432	8.04	7.37	1308	2.93	2.85	1806	2.86	2.70	5934	9.64	8.98
2011	11431	7.84	7.36	9084	10.38	9.50	2347	4.03	3.94	2816	3.82	3.62	8615	11.95	11.14
2012	17162	8.67	8.13	12616	12.56	11.43	4546	4.66	4.52	4097	4.08	3.86	13065	13.37	12.47
2013	23011	10.16	9.48	16628	14.90	13.48	6383	5.56	5.35	5591	4.87	4.60	17420	15.60	14.45
2014	35435	12.29	11.50	25474	17.68	16.12	9961	6.91	6.63	8846	6.05	5.73	26589	18.72	17.38
2015	42249	13.17	12.33	28656	18.59	16.99	13593	8.15	7.77	10178	6.25	5.94	32071	20.28	18.83
2016	50424	13.22	12.40	34425	17.87	16.42	15999	8.47	8.08	12240	6.32	6.02	38184	20.32	18.90
2017	60693	13.91	13.16	40656	19.07	17.74	20037	8.98	8.6	14572	6.59	6.35	46121	21.43	20.08
2018	84593	16.17	15.39	50330	21.32	19.97	34263	11.93	11.47	21198	7.98	7.73	63395	24.6	23.20
Total	351330	11.82	8.69	240788	15.42	11.40	110542	7.84	5.08	85622	5.69	4.18	265708	18.13	13.28

ASIR: age-standardized incidence rate

Table 2 Joinpoint regression analysis results of thyroid cancer incidence rate in China, 2005-2018 (%)

Index	National	Urban	Rural	Male	Female
Year	2005-2018	2005-2010	2005-2018	2005-2018	2005-2018
APC	10.7	10.1	15.2	11.5	10.5
(95%CI)	(9.5, 11.9)	(4.7, 12.5)	(13.7, 16.8)	(10.2, 12.8)	(9.3, 11.7)
Year		2010-2014			
APC		19.0			
(95%CI)		(15.3, 23.9)			
Year		2014-2018			
APC		4.6			
(95%CI)		(-0.1, 7.7)			
AAPC	10.7	11.0	15.2	11.5	10.5
(95%CI)	(9.5, 11.9)	(11.0, 13.8)	(13.7, 16.8)	(10.2, 12.8)	(9.3, 11.7)

AAPC, average annual percentage change; APC, annual percent change

Fig 1 Joinpoint regression in the incidence rate of thyroid cancer in China, 2005-2018.

Age-period-cohort model analysis of thyroid cancer incidence rate in China

From 2005 to 2018, the age effect of thyroid cancer in China exhibited an upward trend followed by a decline at the national level. The incidence rate of thyroid cancer in people aged 0–54 years increased the risk of incidence with age, and the incidence rate in people aged 55–84 years decreased the risk of incidence rate with age. The 50–54 year age group had the highest risk of incidence rate, with age effect coefficients of 1.86 nationwide, 2.00 in males, 1.80 in females, 1.88 in urban areas, and 1.72 in rural areas.

The period effect of thyroid cancer in China, both nationally and across genders and regions, demonstrated an increasing trend during 2005–2015. The national effect coefficient increased from -0.52 to 0.64, from -0.60 to 0.72 in males, from -0.50 to 0.61 in females, from -0.59 to 0.70 in urban areas, and from -0.71 to 0.86 in rural areas.

Among the cohort effects, 1925–1929 had the highest risk of incidence rate, with effect coefficient of 2.06 nationwide, 2.60 in males and 1.84 in females, and 2.20 in urban areas and 2.06 in rural areas. (Fig 2)

Fig 2 Age-period-cohort analysis of national (A), urban (B), rural (C), male (D), and female (E) thyroid cancer in China, 2005-2018

Spatial autocorrelation analysis of thyroid cancer incidence rate in China

During 2010–2016, the high incidence rate of thyroid cancer in China was concentrated in Xinjiang and Inner Mongolia, as well as in eastern and northeastern China, and all provinces showed an increasing trend. (Fig 3)

Global spatial autocorrelation analysis showed that there was a positive spatial autocorrelation and spatial cluster in the incidence rate of thyroid cancer in China during 2010–2016. (Table 3)

To further identify clustered areas, localized spatial autocorrelation analysis of thyroid cancer incidence rate was conducted. The results showed that from 2010 to 2016, there were high-high, low-low, high-low and low-high in some regions. In 2010, there were six high-high cluster regions, and in 2011–2012, the high-high cluster regions expanded to the northeast, and in 2013–2016, the high-high cluster regions were in the northeast. From 2010 to 2011, the number of low-low cluster regions changed from 7 to 9, and in 2012, it was extended to the north-west, and from 2013 to 2016, it moved southward, and finally, there were 12 low-low cluster regions (Fig 4). As a whole, the high-high cluster of thyroid cancer shifted from east China to northeast China, the low-low cluster extended to the south, and the high-low cluster or low-high cluster shifted to the south. The high-low cluster or low-high cluster was less abnormal.

Fig 3 Incidence rates of thyroid cancer in different regions of China, 2010-2016

Table 3 Global Moran's *I* index of thyroid cancer incidence rate in China from 2010 to 2016

Year	Moran's *I* index	Expectation index	Variance	*Z*	*P*
2010	0.391	-0.033	0.008	4.787	0.000
2011	0.520	-0.033	0.008	6.263	0.000
2012	0.621	-0.033	0.008	7.385	0.000
2013	0.468	-0.033	0.008	5.754	0.000
2014	0.476	-0.033	0.007	6.091	0.000
2015	0.429	-0.033	0.007	5.396	0.000
2016	0.407	-0.033	0.007	5.262	0.000

Fig 4 Local spatial autocorrelation of thyroid cancer incidence rate in China, 2010-2016

Discussion

The incidence rate of thyroid cancer in China has shown a sustained increase, with significant heterogeneity across age, sex, and geographic region. The highest incidence rate occurred in the 50–54 year age group. Furthermore, the incidence rate among females was approximately three- to four-fold higher than that among males. In addition, incidence rates were higher in urban areas than in rural areas. Notably, the northeastern region exhibited a high–high cluster of thyroid cancer incidence. These findings provide a solid foundation for developing effective preventive and control measures against thyroid cancer.

The number of new thyroid cancer cases has continued to rise, a trend driven by multiple factors. Obesity represents a major contributing factor. Obesity can lead to metabolic disorders and abnormal hormone secretion, thereby increasing the risk of thyroid cancer. Studies have shown that for every 5kg/m^2 increase in BMI, the risk of thyroid cancer may rise by 6%–25% [32]. According to the Report on Chinese Residents' Nutrition and Chronic Disease Status (2020), the overweight rate among

residents aged 18 and above was 34.3%, an increase of 4.2 percentage points compared with that in 2015 [33]. With advancements in thyroid cancer diagnostic techniques, the early detection rate has significantly improved. Results from a Chinese cohort study showed that the early detection rate of thyroid cancer increased from 29.98% in 2016 to 56.87% in 2020 [34], which may also account for the increase in incidence rate. In addition, at low radiation doses (<0.2 Gray), a linear relationship has been observed between thyroid radiation exposure in children and subsequent thyroid cancer risk. This risk appears 5–10 years after exposure and persists for more than 45 years [35]. The long-term risk accumulation from childhood exposure is an important factor in the current increase in thyroid cancer incidence rate.

Although the incidence rate of thyroid cancer in females was higher than in males, the rate of increase was lower. The higher incidence rate in females may be associated with the histological characteristics of thyroid cancer: estrogen-activated ERα may mediate the growth and malignant progression of thyroid cancer [36]. In addition, overdiagnosis has contributed to the increase incidence of thyroid cancer in females. Among female thyroid cancer patients diagnosed in China between 1998 and 2012, overdiagnosis accounted for approximately 87% of cases [37]. In contrast, the incidence rate in males increased more rapidly, possibly due to the higher prevalence of overweight and obesity among males (41.1% overweight and 18.2% obese), compared with 27.7% and 9.4%, respectively, in females [32].

The incidence rate of thyroid cancer in urban areas was significantly higher than

in rural areas, but the rate of change was lower than in rural areas. Urban residents often experience a fast-paced lifestyle, elevated psychological stress, and frequent sleep deprivation, all of which may contribute to increased thyroid cancer risk. Studies have shown that stress and irritability increase thyroid cancer risk, whereas good sleep quality serves as a protective factor [38]. As the allocation structure of medical resources in rural areas is optimized and the popularization of detection technologies such as high-resolution B-ultrasound, a large number of previously undetected microcarcinomas were identified, leading to a faster growth rate of thyroid cancer incidence rate in rural areas.

Age effect analysis showed that the trend of thyroid cancer incidence rate was basically consistent nationwide, first increasing and then decreasing with age. The age effect coefficient of thyroid cancer was the highest in middle-aged and elderly populations, indicating that the risk of thyroid cancer is the highest in this age group. This may be attributed to two factors: first, childhood exposure to risk factors such as radiation, whose long-term cumulative effects manifest in middle and old age [35]; second, the prevalence of thyroid nodules increases with age with approximately 18.1% shown to be malignant [39, 40], which directly pushes up the incidence rate of thyroid cancer.

The results of the period effect showed that the risk of incidence rate was on the rise, primarily attributable to advancements in diagnostic techniques (such as high-resolution ultrasound) and their widespread clinical application, which enabled the detection of previously occult thyroid cancers [41, 42]. The overall birth cohort

effect showed a downward trend. On the one hand, the improvement of diagnostic capabilities led to a higher early detection rate, which reduced the incidence rate to a certain extent; on the other hand, the health awareness of subsequent birth cohorts has generally increased, and they may reduce the risk of incidence rate through healthier lifestyles or earlier medical intervention [43].

Spatial distribution showed that East China had the highest concentration of thyroid cancer in 2010. This may be due to regional differences in diagnostic levels, with the eastern coastal region having a significantly higher incidence rate of ultrasound screening than other regions due to its developed economy and abundant medical resources [44]. The area of spatial aggregation shifted northwards between 2010 and 2012, with the Northeast region identified as a high–high cluster in 2013 and 2016. This phenomenon may be influenced by dietary habits in the Northeast, which are typically characterized by a high intake of oil, fat, and sugar.

This study has several limitations. Data on thyroid cancer were obtained from the China Cancer Registry Annual Report. However, only case numbers were available, without pathological classification. Furthermore, there was a three-year delay in the latest data, and the number of surveillance sites had increased, resulting in biased results.

Conclusion

From 2005 to 2018, the incidence rate of thyroid cancer in China consistently increased. The northeastern region was identified as a high-incidence hotspot for thyroid cancer. To effectively reduce the risk of thyroid cancer, the national

monitoring system should be strengthened, and a registration system for high-risk populations should be established, accompanied by regular follow-up monitoring to facilitate early detection and timely intervention. More investment should also be made in etiological research to continuously improve the scientific basis of prevention and control efforts. Additionally, measures such as weight control and standardized radiation exposure management should be implemented to reduce the risk of thyroid cancer. These measures, when coordinated with each other, will play a positive role in preventing thyroid cancer.

Author contributions

Conceptualization: Wen Liu and Jiaqi Yang**. Data curation:** Wen Liu and Jiaqi Yang. **Formal analysis:** Wen Liu and Jiaqi Yang. **Investigation:** Wen Liu, Jiaqi Yang, Zhenhao Zheng and Yang Yu. **Methodology:** Wen Liu, Jiaqi Yang, Zhenhao Zheng and Yang Yu. **Software:** Wen Liu, Jiaqi Yang, Zhenhao Zheng and Yang Yu **Supervision:** Zhenhao Zheng and Yang Yu **Writing - original draft:** Wen Liu and Jiaqi Yang. **Writing - review & editing:** Jun Li, Ting Ma and Hao Liu

Acknowledgements

We sincerely thank all the staff involved in the China Cancer Registry Annual Report.

Funding

This work was supported by Bingtuan Science and Technology Programs (2023ZD004,2023ZD009).

Data availability statement

The data is sourced from the China Cancer Registry Annual Report (2008–2021)

released by the National Cancer Center of China. The diagnostic criteria for thyroid cancer utilized in this research are based on the Tenth Revision of the International Classification of Diseases (ICD-10/C73).

Competing interests

The authors declare no competing interests.

References

1. Bray F, Laversanne M, Sung H, Ferlay J, Siegel RL, Soerjomataram I, et al. Global cancer statistics 2022: GLOBOCAN estimates of incidence and mortality worldwide for 36 cancers in 185 countries. CA Cancer J Clin. 2024;74(3). https://doi.org/10.3322/caac.21834.

2. Zhang L, Feng Q, Wang J, Tan Z, Li Q, Ge M. Molecular basis and targeted therapy in thyroid cancer: Progress and opportunities. Biochim Biophys Acta Rev Cancer. 2023;1878(4). https://doi.org/10.1016/j.bbcan.2023.188928.

3. Cancer Today [EB/OL] [2025-04-05] https://gco.iarc.fr/en

4. Huang K, Wang N, Huang X, Qian S, Cai Y, Wu F, et al. Global, regional, and national burden of thyroid cancer in young people aged 10-24 years from 1990 to 2021: an analysis based on the Global Burden of Disease Study 2021. BMC Public Health. 2025;25(1). https://doi.org/10.1186/s12889-025-22322-1.

5. Yin Y, Zhang X. A comprehensive analysis and comparative study of the trends in thyroid cancer burden in China and globally from 1990 to 2021, with projections for the next 15 Years. Front Oncol. 2025;15. https://doi.org/10.3389/fonc.2025.1505728.

6. Dou Z, Shi Y, Jia J. Global burden of disease study analysis of thyroid cancer burden across 204 countries and territories from 1990 to 2019. Frontiers in Oncology. 2024;14. https://doi.org/10.3389/fonc.2024.1412243.

7. Hu X, Lv J, Qin C, Liu X, Zhu J, Cui Y. Global and China burden of hormone-related cancers and risk factors, 1990-2021: results from the Global Burden of Disease Study 2021. BMC Public Health. 2025;25(1). https://doi.org/10.1186/s12889-025-22768-3.

8. W. Chen Z, P. China Cancer Registry Annual Report 2008. Military Medical Science Press; 2009.

9. W. Chen Z, P. China Cancer Registry Annual Report 2009. Beijing: Military Medical Science Press; 2010.

10. W. Chen Z, P. China Cancer Registry Annual Report 2010. Beijing: Military Medical Science Press; 2011.

11. J. He WC. China Cancer Registry Annual Report 2011. Beijing: Military Medical Science Press; 2012.

12. J. He WC. China Cancer Registry Annual Report 2012. Beijing: Military Medical Science Press; 2013.

13. J. He WC. China Cancer Registry Annual Report 2013. Beijing: Military Medical Science Press; 2014.

14. J. He WC. China Cancer Registry Annual Report 2014. Beijing: Military Medical Science Press; 2015.

15. J. He WC. China Cancer Registry Annual Report 2015. Beijing: Military Medical Science Press; 2016.

16. J. He WC. China Cancer Registry Annual Report 2016. Beijing: Military Medical Science Press; 2017.

17. J. He WC. China Cancer Registry Annual Report 2017. Beijing: Military Medical Science Press; 2018.

18. He J. China Cancer Registry Annual Report 2018. Beijing: Military Medical Science Press; 2019.

19. J. He WC. China Cancer Registry Annual Report 2019. Beijing: Military Medical Science Press; 2020.

20. J. He WC. China Cancer Registry Annual Report 2020. Beijing: Military Medical Science Press; 2021.

21. J. He WC. China Cancer Registry Annual Report 2021. Beijing: Military Medical Science Press; 2022.

22. Outland B NM, William MJ. Health Policy Basics: Implementation of the International Classification of Disease, 10th Revision. Ann Intern Med. 2015;163(7). https://doi.org/10.7326/M15-1933.

23. Census data-National Bureau of Statistics [EB/OL] [2025-02-09] https://www.stats.gov.cn/sj/pcsj/

24. Li HZ DL. Application of Joinpoint regression model in cancer epidemiological time trend analysis. Zhonghua Yu Fang Yi Xue Za Zhi. 2020;54(8). https://doi.org/10.3760/cma.j.cn112150-20200616-00889.

25. Kim HJ, Fay MP, Feuer EJ, Midthune DN. Permutation tests for joinpoint regression with applications to cancer rates. Statist Med. 2000;19(3). https://doi.org/10.1002/(sici)1097-0258(20000215)19:3<335::aid-sim336>3.0.co;2-z.

26. Nistal-Nuño B. Joinpoint regression analysis to evaluate traffic public health policies by national temporal trends from 2000 to 2015. International Journal of Injury Control and Safety Promotion. 2017;25(2). https://doi.org/10.1080/17457300.2017.1341937.

27. Fu WJ. Ridge estimator in singulah oesiun with application to age-period-cohort analysis of disease rates. Communications in Statistics - Theory and Methods. 2000;29(2). https://doi.org/10.1080/03610920008832483.

28. YANG Y. adult chronic disease mortality, 1960-1999: age, period, and cohort variations. Demography. 2008;45(2). https://doi.org/10.1353/dem.0.0000.

29. Yang Y. A METHODOLOGICAL COMPARISON OF AGE-PERIOD-COHORT MODELS: THE INTRINSIC ESTIMATOR AND CONVENTIONAL GENERALIZED LINEAR MODELS. Sociological methodology. 2004;34(1). https://doi.org/10.1111/j.0081-1750.2004.00148.x.

30. Mubarik S, Shakil Malik S, Wang Z, Li C, Fawad M, Yu C. Recent insights into breast cancer incidence trends among four Asian countries using age-period-cohort model. Cancer Management and Research. 2019;Volume 11. https://doi.org/10.2147/cmar.S208323.

31. Chen YG. Theoretical development and methodological improvement of spatial autocorrelation based on Moran statistics. Geographical Research. 2009;28(6). https://doi.org/10.11821/yj2009060002.

32. Chen K, Shen Z, Gu W, Lyu Z, Qi X, Mu Y, et al. Prevalence of obesity and associated complications in China: A cross-sectional, real-world study in 15.8 million adults. Diabetes Obes Metab. 2023;25(11). https://doi.org/10.1111/dom.15238.

33. Report on Nutrition and Chronic Disease Status of Chinese Residents (2020). 2025-02-09.

34. Xie S-H, Huang R-Q, Liu Y-L, Cao S-M, Qian C-N. An increase in early cancer detection rates at a single cancer center: Experiences from Sun Yat-sen University Cancer Center. Visualized Cancer Medicine. 2022;3. https://doi.org/10.1051/vcm/2022001.

35. Lubin JH, Adams MJ, Shore R, Holmberg E, Schneider AB, Hawkins MM, et al. Thyroid Cancer Following Childhood Low-Dose Radiation Exposure: A Pooled Analysis of Nine Cohorts. The Journal of Clinical Endocrinology & Metabolism. 2017;102(7). https://doi.org/10.1210/jc.2016-3529.

36. Yanhong Huang WD, Jing Li,Hao Zhang,Zhongyan Shanand Weiping Teng. Differential expression patterns and clinical significance of estrogen receptor-α and β in papillary thyroid carcinoma. BMC Cancer. 2014;14. https://doi.org/10.1186/1471-2407-14-383.

37. Miranda-Filho A, Lortet-Tieulent J, Bray F, Cao B, Franceschi S, Vaccarella S, et al. Thyroid cancer incidence trends by histology in 25 countries: a population-based

study. The Lancet Diabetes & Endocrinology. 2021;9(4). https://doi.org/10.1016/s2213-8587(21)00027-9.

38. Afrashteh S, Fararouei M, Parad MT, Mirahmadizadeh A. Sleep quality, stress and thyroid cancer: a case-control study. J Endocrinol Invest. 2022;45(6). https://doi.org/10.1007/s40618-022-01751-4.

39. Kwong N, Medici M, Angell TE, Liu X, Marqusee E, Cibas ES, et al. The Influence of Patient Age on Thyroid Nodule Formation, Multinodularity, and Thyroid Cancer Risk. The Journal of Clinical Endocrinology & Metabolism. 2015;100(12). https://doi.org/10.1210/jc.2015-3100.

40. Yoon JH. Malignancy Risk Stratification of Thyroid Nodules: Comparison between the Thyroid Imaging Reporting and Data System and the 2014 American Thyroid Association Management Guidelines. Radiology. 2016;278(3). https://doi.org/10.1148/radiol.2015150056.

41. Udelsman R, Zhang Y. The epidemic of thyroid cancer in the United States: the role of endocrinologists and ultrasounds. Thyroid. 2014;24(3). https://doi.org/10.1089/thy.2013.0257.

42. Liu Y, Su L, Xiao H. Review of Factors Related to the Thyroid Cancer Epidemic. Int J Endocrinol. 2017;2017. https://doi.org/10.1155/2017/5308635.

43. Li Y, Lv X, Liang J, Dong H, Chen C. The development and progress of health literacy in China. Front Public Health. 2022;10. https://doi.org/10.3389/fpubh.2022.1034907.

44. Du L, Wang Y, Sun X, Li H, Geng X, Ge M, et al. Thyroid cancer: trends in incidence, mortality and clinical-pathological patterns in Zhejiang Province, Southeast China. BMC Cancer. 2018;18(1). https://doi.org/10.1186/s12885-018-4081-7.

fig 1 Click here to access/download;Figure;fig 1.docx

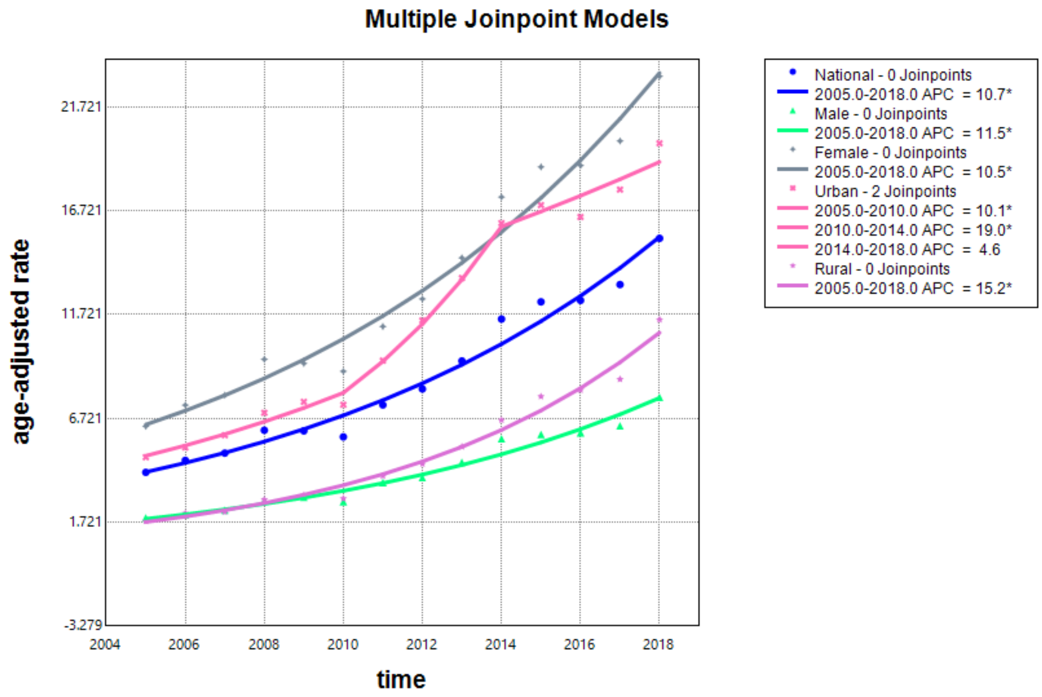

Fig 1 Joinpoint regression in the incidence of thyroid cancer in China, 2005-2018.

Click here to access/download;Figure;fig 2.docx

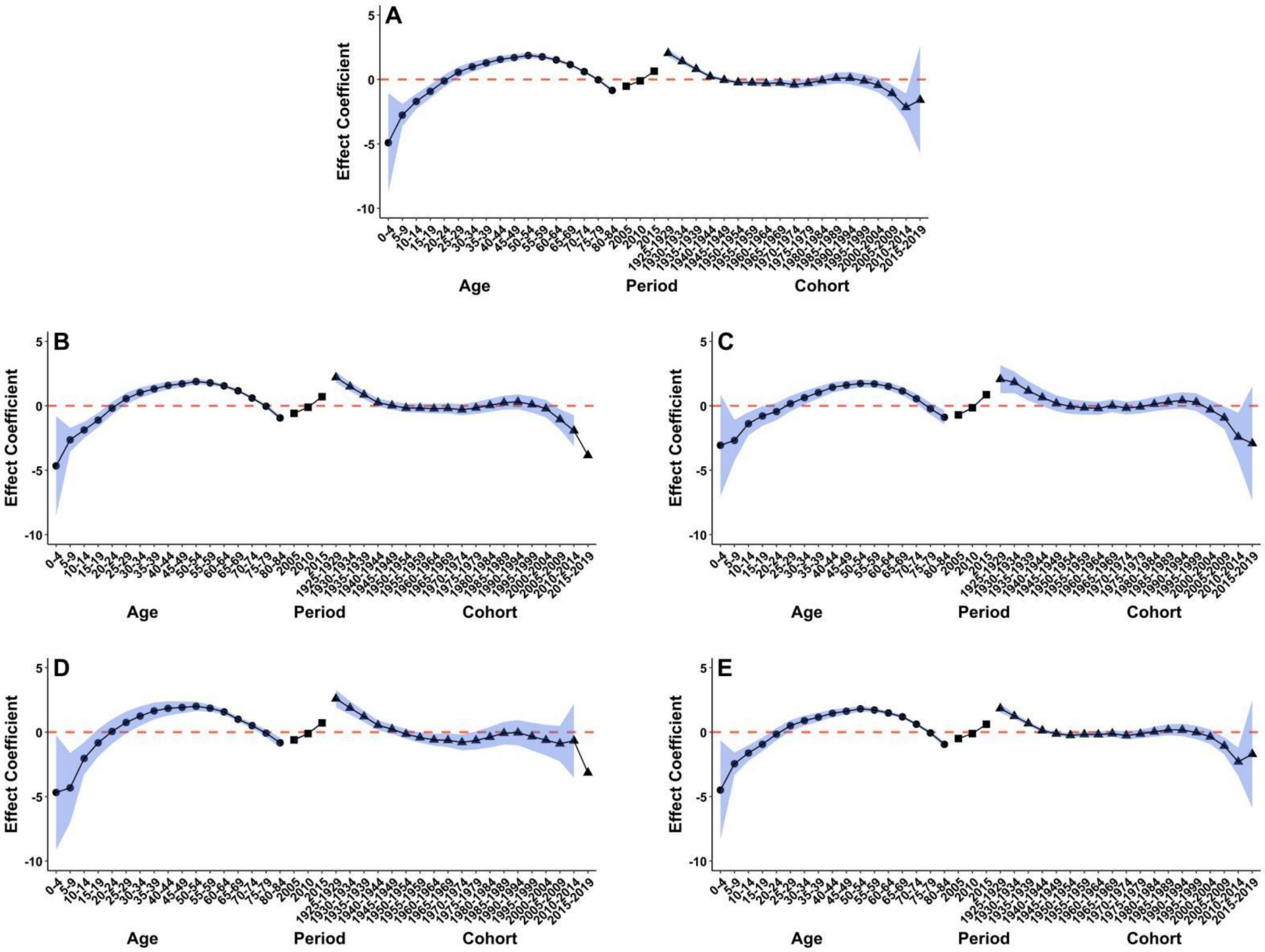

Fig 2 Age-period-cohort analysis of national (**A**), urban (B), rural (**C**), male (**D**), and female (**E**) thyroid cancer in China, 2005-2018

Click here to access/download;Figure;fig 3.docx

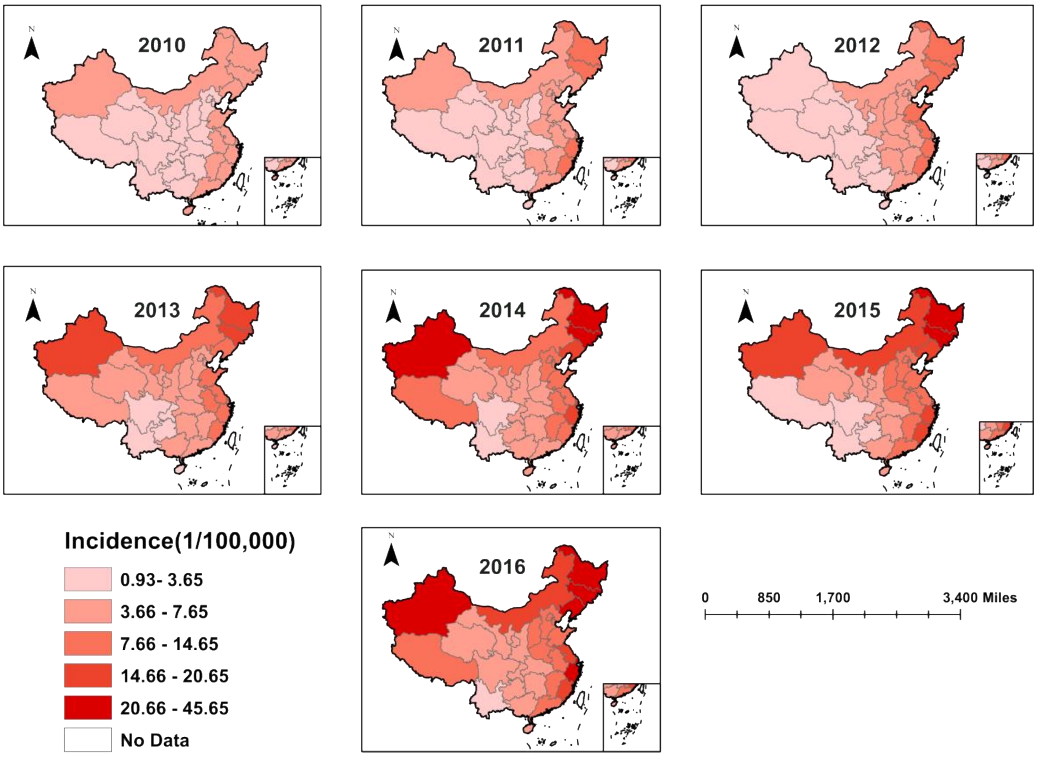

Fig 3 Incidences of thyroid cancer in different regions of China, 2010-2016

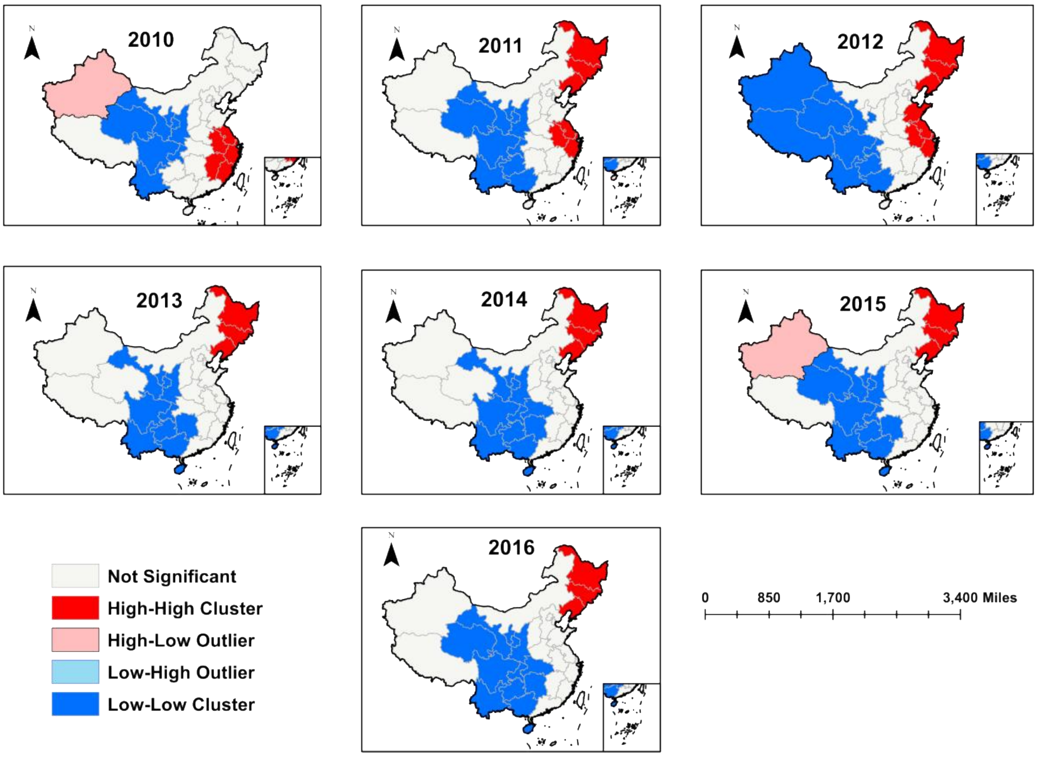

1

2 **Fig 4** Local spatial autocorrelation of thyroid cancer incidence in China, 2010-2016
