# Supplementary material for: Spatial and temporal distribution of thyroid cancer incidence in China: An ecological study based on a national registry
Source: PLoS One. 2026 Mar 20;21(3):e0341734. doi: 10.1371/journal.pone.0341734 (PMC13004386; doi:10.1371/journal.pone.0341734)
Supplement: Attachment — Submitted filename: Review for PLOS 1-10-2025.docx [file pone.0341734.s003.docx]

## Review of Manuscript: -

Reviewer comments for authors: -

## Title & Abstract

Study design not explicitly mentioned, clearly state the design in the title and mention key limitations in the abstract (no histology data, registry variability).

## Introduction

Prior studies in China are briefly noted but not critically compared; objectives are broad.

Strengthen literature review by contrasting with existing Chinese data; clarify that the study is descriptive, aiming to identify spatiotemporal patterns rather than causality.

## Methods

- Study design not clearly declared at the start.
- Start with a clear statement: 'This is a nationwide registry-based descriptive ecological study using cancer registry data.'
- Define inclusion/exclusion criteria for cases.
- Explicitly describe potential biases in data sources and how they were addressed.
- Handling of missing or incomplete data not mentioned.
- State how missing data were handled.
- If sensitivity analyses were not done, acknowledge this limitation.Results
- Incidence rates presented without confidence intervals.
- Add 95% CIs to crude and standardized incidence rates.
- Spatial clustering results shown in maps only, not summarized in tables.
- Provide a supplementary table listing provinces with high–high, low–low, etc. clusters for clarity.Discussion

Expand and structure the limitations section (ecological fallacy, lack of histology and staging data, data lag, registry expansion, possible misclassification).

- Some explanations are speculative without direct data support.
- Temper causal language and clarify that observed patterns are descriptive and hypothesis-generating.

## Other Information

- Ethics marked 'N/A' with no explanation.
- Clarify that ethics approval was not required as de-identified secondary registry data were used, or specify if a waiver was granted.
