# Supplementary material for: Spatial and temporal distribution of thyroid cancer incidence in China: An ecological study based on a national registry
Source: PLoS One. 2026 Mar 20;21(3):e0341734. doi: 10.1371/journal.pone.0341734 (PMC13004386; doi:10.1371/journal.pone.0341734)
Supplement: Attachment — Submitted filename: Response to Editors and Reviewers .docx [file pone.0341734.s005.docx]

PLOS One

November 30, 2025

Manuscript Number: PONE-D-25-46157

"Spatial and temporal distribution of thyroid cancer incidence in China: A study based on a national registry"

**Dear Editor and Reviewers:**

We are very grateful to receive comments on the revision of the manuscript entitled "Spatial and temporal distribution of thyroid cancer incidence in China: A study based on a national registry" (ID: PONE-D-25-46157). We appreciate for editors and reviewers' warm work earnestly.

We sincerely thank all editors and reviewers for their valuable feedback that we have used to clarify the thinking of the study, make the structure of the study more reasonable, and finally improve the quality of our manuscript.

The reviewer comments are laid out below in italic font and specific concerns have been numbered. Our response is given in normal font and changes/additions to the manuscript are given in the blue text.

On the separate pages, we provided our response to the comments and suggestions, point by point, and highlighted the changes in the marked copy of the revision. We hope that our revision will be approved by the experts and reviewed favorably.

Sincerely,

Hao Liu,

Reviewer Comments:

**Reviewer 1**

1. *Dear Author, The article is a bit lengthy. Please ensure that it is precise and comprehensible specially the statistical indicators. Please adhere to the guidelines for authors as per PLOS One requirements.*

**Response:** Thank you very much for your careful review. We have provided an accurate description of the entire text, especially reorganized the methods section to make the paper more logically structured. The modifications have left their marks in the text and we hope for your approval.

**Reviewer 2**

*1. Title & Abstract
Study design not explicitly mentioned, clearly state the design in the title and mention key limitations in the abstract (no histology data, registry variability).*

**Response:** We sincerely thank you for your valuable time and insightful comments on our manuscript. We have revised the Title: “Spatial and temporal distribution of thyroid cancer incidence in China: An ecological study based on a national registry”.

Furthermore, we have added a concise statement on the key limitations directly within the abstract (Page 2-3, Line 21-27):

"Although this study is limited by the lack of pathological classification, the delay in the latest data, and potential biases from the increasing number of surveillance sites, these findings also suggest that the national thyroid cancer surveillance system should be further strengthened, a registration system for high-risk populations should be established, and greater investment should be allocated to etiological research, such as through measures like controlling weight and regulating radiation to reduce the risk of thyroid cancer."

*2. Introduction
2.1 Prior studies in China are briefly noted but not critically compared; objectives are broad.*

**Response:** We are deeply grateful for your insightful feedback regarding the need for a more critical discussion of prior research and more precisely defined objectives. We have added (Page 4-5, Line 48-54):

**"**However, at present, there is a lack of comprehensive studies on the spatial and temporal distribution of thyroid cancer incidence in China. Some studies are limited to specific geographical areas or populations, lacking representativeness on a national scale [8]. Additionally, although other studies have described the overall trend of thyroid cancer incidence in China, they have not systematically revealed the regional clustering characteristics of thyroid cancer incidence from a national perspective [9-11]. "

Furthermore, we have refined the statement of our research objectives (Page 5, Line 60-65):

**"These analyses will help to gain a deeper understanding of the temporal and spatial distribution of thyroid cancer in China and its epidemiological trends, identify key areas for precise prevention and public health intervention of thyroid cancer, provide data to support long-term prevention and treatment efforts for thyroid cancer, and offer a reference for other countries around the world.**"

*2.2 Strengthen literature review by contrasting with existing Chinese data; clarify that the study is descriptive, aiming to identify spatiotemporal patterns rather than causality.*

**Response:** We sincerely thank you for your valuable feedback. We have added (Page 4-5, Line 48-54):

**"**However, at present, there is a lack of comprehensive studies on the spatial and temporal distribution of thyroid cancer incidence in China. Some studies are limited to specific geographical areas or populations, lacking representativeness on a national scale [8]. Additionally, although other studies have described the overall trend of thyroid cancer incidence in China, they have not systematically revealed the regional clustering characteristics of thyroid cancer incidence from a national perspective [9-11]. "

Page 5, Line 55-65:

"Comprehensive understanding of the epidemic characteristics of diseases is a key prerequisite for establishing an effective prevention and control system. As an ecological study, based on 14–years of data from the China Cancer Registry Annual Report from 2008 to 2021, this study described the epidemic characteristics of thyroid cancer incidence and its age-period-cohort effects, and used spatial autocorrelation methods to describe the spatial distribution characteristics. These analyses will help to gain a deeper understanding of the temporal and spatial distribution of thyroid cancer in China and its epidemiological trends, identify key areas for precise prevention and public health intervention of thyroid cancer, provide data to support long-term prevention and treatment efforts for thyroid cancer, and offer a reference for other countries around the world."

*3. Methods
3.1 Study design not clearly declared at the start.*

**Response:** We sincerely thank you for this important observation. We have added (Page 5, Line 68-69):

" This is a descriptive ecological study based on the China Cancer Registry Annual Report." *3.2 Start with a clear statement: 'This is a nationwide registry-based descriptive ecological study using cancer registry data.'*

**Response:** We appreciate your specific suggestion. We have added the sentence (Page 5, Line 68-69):

" This is a descriptive ecological study based on the China Cancer Registry Annual Report." *3.3 Define inclusion/exclusion criteria for cases.*

**Response:** We sincerely thank you for this important comment. Based on the information processed from the annual report data, we have added a description of data quality control in the methods section. We directly used the data from the annual report, and the report directly compiled and statistically analyzed the cancer incidence data for different regions and genders, etc. There were no additional personal information or variables, and no issues related to handling missing data were involved. We hope you can understand and approve of this.

(Page 6-7, Line 77-98):

"**Data Quality**

To ensure the authenticity, stability, and comparability of the data, the National Cancer Center implemented rigorous quality control procedures. This process included checks for completeness, validity verification, and logical consistency assessments. Any identified issues, such as missing or inconsistent entries, were returned to the local registries for verification and correction.

The inclusion and exclusion of data strictly adhered to the quality control standards for Chinese cancer registration work. Data included in the report must meet the following core quality requirements: the morphological verification percentage (MV%) should range between 55% and 95%; the percentage of death certificate only (DCO%) should be less than 20%; the mortality-to-incidence ratio (M/I) should fall within the range of 0.55 to 0.85; the cancer trend should be relatively stable, the level reasonable, and the mortality rate no lower than 100/100,000.

Nevertheless, potential biases persist in China's thyroid cancer registration data. First, although the data are derived from a nationwide cancer registration system, variations in the number of national-level registries included across different years may affect the representativeness of the data. Second, historically limited healthcare resources in rural areas have resulted in significant disparities in diagnostic capacity, which impacts the completeness of case registration. Finally, possible duplication or omissions in data reporting may compromise data accuracy. However, it has been confirmed that the thyroid cancer incidence data from 2005 to 2018 consistently met quality standards (Table 1)."

*3.4 Explicitly describe potential biases in data sources and how they were addressed.*

**Response:** We sincerely thank you for this valuable comment. We have added the following text (Page 6-7, Line 77-101):

"**Data Quality**

To ensure the authenticity, stability, and comparability of the data, the National Cancer Center implemented rigorous quality control procedures. This process included checks for completeness, validity verification, and logical consistency assessments. Any identified issues, such as missing or inconsistent entries, were returned to the local registries for verification and correction.

The inclusion and exclusion of data strictly adhered to the quality control standards for Chinese cancer registration work. Data included in the report must meet the following core quality requirements: the morphological verification percentage (MV%) should range between 55% and 95%; the percentage of death certificate only (DCO%) should be less than 20%; the mortality-to-incidence ratio (M/I) should fall within the range of 0.55 to 0.85; the cancer trend should be relatively stable, the level reasonable, and the mortality rate no lower than 100/100,000.

Nevertheless, potential biases persist in China's thyroid cancer registration data. First, although the data are derived from a nationwide cancer registration system, variations in the number of national-level registries included across different years may affect the representativeness of the data. Second, historically limited healthcare resources in rural areas have resulted in significant disparities in diagnostic capacity, which impacts the completeness of case registration. Finally, possible duplication or omissions in data reporting may compromise data accuracy. However, it has been confirmed that the thyroid cancer incidence data from 2005 to 2018 consistently met quality standards (Table 1)."

**Table 1** Quality evaluation of thyroid cancer data in China from 2005 to 2018

| **Year** | **National** | | |  | **Urban** | | |  | **Rural** | | |
| --- | --- | --- | --- | --- | --- | --- | --- | --- | --- | --- | --- |
|  | **MV(%)** | **DCO(%)** | **M/I** |  | **MV(%)** | **DCO(%)** | **M/I** |  | **MV(%)** | **DCO(%)** | **M/I** |
| 2005 | 89.22 | 0.34 | 0.11 |  | 90.15 | 0.38 | 0.09 |  | 80.77 | 0.00 | 0.23 |
| 2006 | 89.96 | 0.34 | 0.07 |  | 90.55 | 0.30 | 0.07 |  | 84.29 | 0.71 | 0.14 |
| 2007 | 90.44 | 0.19 | 0.08 |  | 90.49 | 0.21 | 0.07 |  | 90.03 | 0.00 | 0.17 |
| 2008 | 89.33 | 0.32 | 0.07 |  | 89.32 | 0.32 | 0.07 |  | 89.50 | 0.25 | 0.12 |
| 2009 | 89.73 | 0.37 | 0.08 |  | 90.13 | 0.34 | 0.08 |  | 87.51 | 0.58 | 0.11 |
| 2010 | 90.25 | 0.32 | 0.07 |  | 92.67 | 0.29 | 0.07 |  | 83.18 | 0.42 | 0.09 |
| 2011 | 91.78 | 0.19 | 0.07 |  | 93.60 | 0.12 | 0.06 |  | 84.70 | 0.47 | 0.11 |
| 2012 | 91.63 | 0.23 | 0.06 |  | 93.29 | 0.17 | 0.05 |  | 87.02 | 0.37 | 0.10 |
| 2013 | 92.14 | 0.13 | 0.05 |  | 94.01 | 0.11 | 0.04 |  | 87.28 | 0.17 | 0.07 |
| 2014 | 91.74 | 0.13 | 0.05 |  | 93.06 | 0.15 | 0.04 |  | 88.34 | 0.10 | 0.07 |
| 2015 | 93.14 | 0.11 | 0.04 |  | 94.65 | 0.08 | 0.04 |  | 89.96 | 0.15 | 0.06 |
| 2016 | 91.52 | 0.09 | 0.04 |  | 93.01 | 0.09 | 0.04 |  | 88.32 | 0.09 | 0.06 |
| 2017 | 89.88 | 0.25 | 0.04 |  | 92.04 | 0.32 | 0.04 |  | 85.62 | 0.12 | 0.06 |
| 2018 | 91.87 | 0.06 | 0.04 |  | 93.29 | 0.06 | 0.03 |  | 89.78 | 0.07 | 0.05 |

MV%, morphological verification percentage; DCO%, percentage of death certificate only;

M/I, mortality-to-incidence ratio.

*3.5 Handling of missing or incomplete data not mentioned./State how missing data were handled*

**Response:** We sincerely thank you for raising this important point. Based on the information processed from the annual report data, we have added a description of data quality control in the methods section. We directly used the data from the annual report, and the report directly compiled and statistically analyzed the cancer incidence data for different regions and genders, etc. There were no additional personal information or variables, and no issues related to handling missing data were involved. We hope you can understand and approve of this.

(Page 6-7, Line 77-98):

"**Data Quality**

To ensure the authenticity, stability, and comparability of the data, the National Cancer Center implemented rigorous quality control procedures. This process included checks for completeness, validity verification, and logical consistency assessments. Any identified issues, such as missing or inconsistent entries, were returned to the local registries for verification and correction.

The inclusion and exclusion of data strictly adhered to the quality control standards for Chinese cancer registration work. Data included in the report must meet the following core quality requirements: the morphological verification percentage (MV%) should range between 55% and 95%; the percentage of death certificate only (DCO%) should be less than 20%; the mortality-to-incidence ratio (M/I) should fall within the range of 0.55 to 0.85; the cancer trend should be relatively stable, the level reasonable, and the mortality rate no lower than 100/100,000.

Nevertheless, potential biases persist in China's thyroid cancer registration data. First, although the data are derived from a nationwide cancer registration system, variations in the number of national-level registries included across different years may affect the representativeness of the data. Second, historically limited healthcare resources in rural areas have resulted in significant disparities in diagnostic capacity, which impacts the completeness of case registration. Finally, possible duplication or omissions in data reporting may compromise data accuracy. However, it has been confirmed that the thyroid cancer incidence data from 2005 to 2018 consistently met quality standards (Table 1)."

*3.6 If sensitivity analyses were not done, acknowledge this limitation.*

**Response:** We sincerely thank you for this suggestion. We have incorporated a statement to this effect into the Limitations of the Discussion (Page 23, Line 355-357):

"Finally, due to the lack of more variable information, we did not conduct any further analyses, including sensitivity analysis." *4. Results
4.1 Incidence rates presented without confidence intervals./Add 95% CIs to crude and standardized incidence rates.*

**Response:** We sincerely thank you for valuable comments. The 95% confidence intervals has been added (Page 9-10, Line 119-125):

"The 95% confidence interval (CI) for crude incidence rate was computed using the exact Poisson method [28]. The 95% CI for the ASIR was estimated using the Fay-Feuer method, which is robust for directly standardized rates across varying population sizes and incidence levels [29].

$$\text{I}\text{ncidence}\text{ }\text{rate}\text{ 95\%}\text{C}\text{I}_{\text{low}}\text{=}\frac{\frac{\text{1}}{\text{2}}\text{(}\text{C}\text{h}\text{iInv}\text{(}\frac{0.05}{\text{2}}\text{,2×}\text{count}\text{))}}{\text{population}}\text{×100,000}$$

$$\text{I}\text{ncidence}\text{ }\text{rate}\text{ 95\%}\text{C}\text{I}_{\text{h}\text{ig}\text{h}}\text{=}\frac{\frac{\text{1}}{\text{2}}\text{(}\text{C}\text{h}\text{iInv}\text{(1−}\frac{0.05}{\text{2}}\text{,2×(}\text{count}\text{+1)))}}{\text{population}}\text{×100,000}$$

$$\text{ASIR}\text{ 95\%}\text{C}\text{I}_{\text{low}}\text{=}\left( \frac{\text{v}}{\text{2×}\text{ASIR}} \right)\text{×}\left( \text{C}\text{h}\text{iInv}\left( \frac{0.05}{\text{2}}\text{,}\frac{\text{(2×}\mathrm{ASIR}^{\text{2}}\text{)}}{\text{v}} \right) \right)\text{×100,000}$$

$$\text{ASIR}\text{ 95\%}\text{C}\text{I}_{\text{h}\text{ig}\text{h}}\text{=}\left( \frac{\text{v}\text{+}\text{w}_{\text{m}}^{\text{2}}}{\text{2×(}\text{ASIR}\text{+}\text{w}_{\text{m}}\text{)}} \right)\text{×}\left( \text{C}\text{h}\text{iInv}\left( \text{1−}\frac{0.05}{\text{2}}\text{,}\frac{\text{2(}\text{ASIR}\text{+}\text{w}_{\text{m}}\text{)}^{\text{2}}}{\text{v}\text{+}\text{w}_{\text{m}}^{\text{2}}} \right) \right)\text{×100,000}$$

Where, $\text{w}_{\text{m}}$ is the weight correction factor, $\text{v}$ is the estimated variance of the ASIR, *ChiInv* (p, n) is the inverse of the chi-squared distribution function evaluated at p and with n degrees of freedom."

(Page 14-15, Line 212-230):

**" Incidence rate and its trends of thyroid cancer in China**

From 2005 to 2018, there were a total of 351,330 cases of thyroid cancer in China, of which 240,788 (68.54%) were in urban areas and 110,542 (31.46%) were in rural areas, with 85,622 (24.37%) in males and 265,708 (75.63%) in females. The incidence rate of thyroid cancer in China was 11.82 (95% CI: 11.79, 11.86) per 100,000, of which 15.42 (95% CI: 15.36, 15.49) per 100,000 were urban areas, 7.84 (95% CI: 7.79, 7.89) per 100,000 were rural areas, 5.69 (95% CI: 5.65, 5.72) per 100,000 were males, and 18.13 (95% CI: 18.06, 18.20) per 100,000 were females. The ASIR was 8.69 (95% CI: 8.55, 8.83) per 100,000, of which 11.40 (95% CI: 11.20, 11.60) per 100,000 were urban areas, 5.08 (95% CI: 4.91, 5.26) per 100,000 were rural areas, 4.18 (95% CI: 4.05, 4.32) per 100,000 were males, and 13.28 (95% CI: 13.02, 13.51) per 100,000 were females.

**Table 1 Incidence rate of thyroid cancer in China, 2005-2018 (1/100,000)**

| Year | National | | |  | Urban | | |  | Rural | | |  | Male | | |  | Female | | | |
| --- | --- | --- | --- | --- | --- | --- | --- | --- | --- | --- | --- | --- | --- | --- | --- | --- | --- | --- | --- | --- |
|  | Case | Rate  (95%CI) | ASIR  (95%CI) |  | Case | Rate  (95%CI) | ASIR  (95%CI) |  | Case | Rate  (95%CI) | ASIR  (95%CI) |  | Case | Rate  (95%CI) | ASIR  (95%CI) |  | Case | Rate  (95%CI) | ASIR  (95%CI) |  |
| 2005 | 2361 | 4.30  (4.13, 4.48) | 4.11  (3.94, 4.28) |  | 2122 | 5.22  (5.00, 5.44) | 4.84  (4.63, 5.05) |  | 239 | 1.68  (1.47, 1.90) | 1.76  (1.54, 2) |  | 550 | 1.98  (1.81, 2.15) | 1.92  (1.77, 2.1) |  | 1811 | 6.68  (6.38, 7.00) | 6.33  (6.04, 6.64) | |
| 2006 | 2977 | 5.00  (4.82, 5.18) | 4.69  (4.52, 4.87) |  | 2697 | 5.79  (5.58, 6.02) | 5.31  (5.11, 5.52) |  | 280 | 2.15  (1.91, 2.42) | 2.14  (1.89, 2.42) |  | 659 | 2.2  (2.03, 2.37) | 2.07  (1.91, 2.24) |  | 2318 | 7.84  (7.53, 8.17) | 7.35  (7.05, 7.66) | |
| 2007 | 3212 | 5.37  (5.19, 5.56) | 5.04  (4.86, 5.22) |  | 2891 | 6.48  (6.25, 6.72) | 5.90  (5.69, 6.13) |  | 321 | 2.11  (1.89, 2.36) | 2.21  (1.98, 2.47) |  | 732 | 2.42  (2.25, 2.60) | 2.29  (2.12, 2.46) |  | 2480 | 8.38  (8.06, 8.72) | 7.83  (7.52, 8.15) | |
| 2008 | 4435 | 6.71  (6.51, 6.91) | 6.14  (5.96, 6.33) |  | 4035 | 7.74  (7.50, 7.98) | 6.98  (6.76, 7.2) |  | 400 | 2.86  (2.59, 3.16) | 2.80  (2.53, 3.09) |  | 993 | 2.98  (2.80, 3.17) | 2.76  (2.59, 2.94) |  | 3442 | 10.49  (10.15, 10.85) | 9.56  (9.24, 9.89) | |
| 2009 | 5607 | 6.56  (6.39, 6.73) | 6.11  (5.95, 6.27) |  | 4742 | 8.25  (8.02, 8.49) | 7.51  (7.3, 7.73) |  | 865 | 3.09  (2.89, 3.30) | 3.02  (2.82, 3.23) |  | 1344 | 3.11  (2.94, 3.28) | 2.91  (2.76, 3.08) |  | 4263 | 10.09  (9.79, 10.40) | 9.36  (9.08, 9.65) | |
| 2010 | 7740 | 6.21  (6.07, 6.35) | 5.82  (5.69, 5.95) |  | 6432 | 8.04  (7.85, 8.24) | 7.37  (7.19, 7.56) |  | 1308 | 2.93  (2.77, 3.09) | 2.85  (2.7, 3.01) |  | 1806 | 2.86  (2.73, 3.00) | 2.70  (2.58, 2.83) |  | 5934 | 9.64  (9.39, 9.89) | 8.98  (8.6, 9.02) | |
| 2011 | 11431 | 7.84  (7.70, 7.99) | 7.36  (7.22, 7.49) |  | 9084 | 10.38  (10.17, 10.59) | 9.50  (9.3, 9.7) |  | 2347 | 4.03  (3.87, 4.20) | 3.94  (3.78, 4.1) |  | 2816 | 3.82  (3.68, 3.97) | 3.62  (3.49, 3.76) |  | 8615 | 11.95  (11.70, 12.20) | 11.14  (10.91, 11.38) | |
| 2012 | 17162 | 8.67  (8.54, 8.80) | 8.13  (8.01, 8.25) |  | 12616 | 12.56  (12.34, 12.78) | 11.43  (11.23, 11.63) |  | 4546 | 4.66  (4.52, 4.79) | 4.52  (4.39, 4.65) |  | 4097 | 4.08  (3.96, 4.21) | 3.86  (3.74, 3.98) |  | 13065 | 13.37  (13.15, 13.61) | 12.47  (12.25, 12.68) | |
| 2013 | 23011 | 10.16  (10.03, 10.29) | 9.48  (9.36, 9.61) |  | 16628 | 14.90  (14.67, 15.13) | 13.48  (13.27, 13.69) |  | 6383 | 5.56  (5.42, 5.69) | 5.35  (5.22, 5.49) |  | 5591 | 4.87  (4.74, 5.00) | 4.60  (4.48, 4.72) |  | 17420 | 15.60  (15.37, 15.84) | 14.45  (14.23, 14.66) | |
| 2014 | 35435 | 12.29  (12.17, 12.42) | 11.50  (11.38, 11.62) |  | 25474 | 17.68  (17.47, 17.90) | 16.12  (15.92, 16.32) |  | 9961 | 6.91  (6.77, 7.05) | 6.63  (6.5, 6.76) |  | 8846 | 6.05  (5.93, 6.18) | 5.73  (5.61, 5.85) |  | 26589 | 18.72  (18.50, 18.95) | 17.38  (17.17, 17.6) | |
| 2015 | 42249 | 13.17  (13.04, 13.29) | 12.33  (12.21, 12.45) |  | 28656 | 18.59  (18.38, 18.81) | 16.99  (16.79, 17.19) |  | 13593 | 8.15  (8.01, 8.29) | 7.77  (7.64, 7.91) |  | 10178 | 6.25  (6.13, 6.38) | 5.94  (5.82, 6.06) |  | 32071 | 20.28  (20.06, 20.50) | 18.83  (18.62, 19.04) | |
| 2016 | 50424 | 13.22  (13.10, 13.33) | 12.40  (12.29, 12.51) |  | 34425 | 17.87  (17.68, 18.06) | 16.42  (16.25, 16.6) |  | 15999 | 8.47  (8.34, 8.60) | 8.08  (7.95, 8.2) |  | 12240 | 6.32  (6.21, 6.43) | 6.02  (5.91, 6.13) |  | 38184 | 20.32  (20.11, 20.52) | 18.90  (18.71, 19.09) | |
| 2017 | 60693 | 13.91  (13.80, 14.02) | 13.16  (13.06, 13.27) |  | 40656 | 19.07  (18.88, 19.25) | 17.74  (17.56, 17.91) |  | 20037 | 8.98  (8.86, 9.11) | 8.6  (8.48, 8.72) |  | 14572 | 6.59  (6.48, 6.70) | 6.35  (6.25, 6.46) |  | 46121 | 21.43  (21.24, 21.63) | 20.08  (19.89, 20.26) | |
| 2018 | 84593 | 16.17  (16.06, 16.28) | 15.39  (15.29, 15.5) |  | 50330 | 21.32  (21.14, 21.51) | 19.97  (19.8, 20.15) |  | 34263 | 11.93  (11.81, 12.06) | 11.47  (11.35, 11.59) |  | 21198 | 7.98  (7.88, 8.09) | 7.73  (7.62, 7.84) |  | 63395 | 24.6  (24.41, 24.80) | 23.20  (23.02, 23.39) | |
| Total | 351330 | 11.82  (11.79, 11.86) | 8.69  (8.55, 8.83) |  | 240788 | 15.42  (15.36, 15.49) | 11.40  (11.20, 11.60) |  | 110542 | 7.84  (7.79, 7.89) | 5.08  (4.91, 5.26) |  | 85622 | 5.69  (5.65, 5.72) | 4.18  (4.05, 4.32) |  | 265708 | 18.13  (18.06, 18.20) | 13.28  (13.02, 13.51) | |

ASIR: age-standardized incidence rate

*4.2 Spatial clustering results shown in maps only, not summarized in tables./Provide a supplementary table listing provinces with high–high, low–low, etc. clusters for clarity*

**Response:** We sincerely thank you for this valuable suggestion. To enhance the clarity of the spatial clustering patterns, we have provided a table (**Table 5**, Page 19):

**Table 5 The clustering pattern of thyroid cancer incidence in China, 2010-2016**

| Year | High-High | High-Low | Low-High | Low-Low |
| --- | --- | --- | --- | --- |
| 2010 | Fujian; Jiangxi; Anhui;  Zhejiang; Jiangsu; Shanghai | Xinjiang |  | Qinghai; Gansu; Ningxia; Shaanxi; Sichuan; Yunnan; Chongqing |
| 2011 | Anhui; Zhejiang; Jiangsu;  Shanghai; Heilongjiang;  Jilin; Liaoning |  |  | Qinghai; Gansu; Ningxia; Shaanxi; Sichuan; Yunnan; Chongqing;  Guizhou; Guangxi |
| 2012 | Anhui; Zhejiang; Jiangsu;  Shanghai; Heilongjiang;  Jilin; Liaoning; Shandong; |  |  | Qinghai; Gansu; Sichuan; Yunnan; Chongqing; Guizhou; Guangxi;  Xinjiang; Xizang |
| 2013 | Heilongjiang; Jilin; Liaoning |  |  | Gansu; Ningxia; Shaanxi; Sichuan; Yunnan; Chongqing; Guizhou; Guangxi; Hunan; Hainan |
| 2014 | Heilongjiang; Jilin; Liaoning |  |  | Gansu; Ningxia; Shaanxi; Sichuan; Yunnan; Chongqing; Guizhou;  Guangxi; Hunan; Hubei; Hainan |
| 2015 | Heilongjiang; Jilin; Liaoning | Xinjiang |  | Gansu; Ningxia; Shaanxi; Sichuan; Yunnan; Chongqing; Guizhou;  Guangxi; Hunan; Qinghai; Hainan |
| 2016 | Heilongjiang; Jilin; Liaoning |  |  | Gansu; Ningxia; Shannxi; Sichuan; Yunnan; Chongqing; Guizhou;  Guangxi; Hunan; Qinghai; Hubei;  Hainan |

*5. Discussion
5.1 Expand and structure the limitations section (ecological fallacy, lack of histology and staging data, data lag, registry expansion, possible misclassification).*

**Response:** We sincerely thank you for this suggestion. We have added (Page 23, Line 347-357):

"This study had several limitations. First, this study was an ecological study conducted at the population level and may be subject to ecological fallacy. This study utilized data from the China Cancer Registry Annual Report, which contained only the number of thyroid cancer cases without details on pathological subtypes, tumor size, or disease stage. The lack of such information made it impossible to differentiate trends between subtypes or to precisely evaluate the degree of overdiagnosis and its contribution to the increasing incidence, thereby limiting a more profound analysis of the trends. Furthermore, the latest data had a three-year delay, and the number of monitoring points has increased, which may lead to bias in the results. Finally, due to the lack of more variable information, we did not conduct any further analyses, including sensitivity analysis."

*5.2 Some explanations are speculative without direct data support.*

**Response:** We sincerely thank you for your valuable comment. We have added.

(Page 20-21, Line 284-305):

The number of new thyroid cancer cases continued to rise, a trend driven by multiple factors. With advancements in thyroid cancer diagnostic techniques, the early detection rate has significantly improved. Results from a Chinese cohort study showed that the early detection rate of thyroid cancer increased from 29.98% in 2016 to 56.87% in 2020 [40]. However, these technological advances may also lead to a certain degree of overdiagnosis, resulting in the earlier detection of asymptomatic and non-lethal small lesions in the thyroid [41]. To address this issue, the China Guidelines for the diagnosis and treatment of thyroid cancer (2022) have stringently restricted biopsy indications and introduced active surveillance with close follow-up for low-risk micro papillary carcinoma [42]. Previous studies showed that both the incidence and mortality of thyroid cancer in China exhibited an upward trend [43]. This key evidence suggested that the increase in thyroid cancer incidence could not be entirely attributed to overdiagnosis, and there might be other risk factors contributing to the rise. Obesity is a probable factor. Studies have shown that for every 5kg/m² increase in body mass index, the risk of thyroid cancer may rise by 6%–25% [44]. According to the Report on Chinese Residents' Nutrition and Chronic Disease Status (2020), the overweight rate among residents aged 18 and above was 34.3%, an increase of 4.2 percentage points compared with that in 2012 [45]. This was consistent with the trends we uncovered in our research. In addition, at low radiation doses (<0.2 Gray), a linear relationship was observed between thyroid radiation exposure in children and subsequent thyroid cancer risk. This risk may appear 5–10 years after exposure and persists for more than 45 years [46].

(Page 21-22, Line 315-324):

"The incidence rate of thyroid cancer in urban areas was significantly higher than in rural areas. Studies have shown that stress and irritability may increase thyroid cancer risk, whereas good sleep quality serves as a protective factor [49]. A cross-sectional study conducted in China showed that urban residents exhibited a significantly higher insomnia rate (19.73%) compared to rural residents (16.99%) [50]. In contrast, the incidence of thyroid cancer in rural areas increased at a faster rate. With the improvement of the New Rural Cooperative Medical System (NRCMS) and the enhancement of its coverage and benefits, the economic barriers to medical care for rural residents were greatly reduced. Previous studies have shown that the coverage rate of the NRCMS increased from 75.20% in 2004 to 94.19% [51]."

*5.3 Temper causal language and clarify that observed patterns are descriptive and hypothesis-generating.*

**Response:** We sincerely thank you for the valuable comments from you. We made rigorous revisions to the discussion section (Page 20-23, Line 284-346):

" The number of new thyroid cancer cases continued to rise, a trend driven by multiple factors. With advancements in thyroid cancer diagnostic techniques, the early detection rate has significantly improved. Results from a Chinese cohort study showed that the early detection rate of thyroid cancer increased from 29.98% in 2016 to 56.87% in 2020 [40]. However, these technological advances may also lead to a certain degree of overdiagnosis, resulting in the earlier detection of asymptomatic and non-lethal small lesions in the thyroid [41]. To address this issue, the China Guidelines for the diagnosis and treatment of thyroid cancer (2022) have stringently restricted biopsy indications and introduced active surveillance with close follow-up for low-risk micro papillary carcinoma [42]. Previous studies showed that both the incidence and mortality of thyroid cancer in China exhibited an upward trend [43]. This key evidence suggested that the increase in thyroid cancer incidence could not be entirely attributed to overdiagnosis, and there might be other risk factors contributing to the rise. Obesity is a probable factor. Studies have shown that for every 5kg/m² increase in body mass index, the risk of thyroid cancer may rise by 6%–25% [44]. According to the Report on Chinese Residents' Nutrition and Chronic Disease Status (2020), the overweight rate among residents aged 18 and above was 34.3%, an increase of 4.2 percentage points compared with that in 2012 [45]. This was consistent with the trends we uncovered in our research. In addition, at low radiation doses (<0.2 Gray), a linear relationship was observed between thyroid radiation exposure in children and subsequent thyroid cancer risk. This risk may appear 5–10 years after exposure and persists for more than 45 years [46].

Our study indicated that thyroid cancer exhibited distinct gender-related patterns: females had a higher overall incidence. Estrogen-activated ERα may mediate the growth and malignant progression of thyroid cancer [47]. Among female thyroid cancer patients diagnosed in China between 1998 and 2012, overdiagnosis accounted for approximately 87% of cases [48]. In contrast, the incidence of thyroid cancer in men increased at a faster rate. The Report on Chinese Residents' Nutrition and Chronic Disease Status indicated a consistent trend of higher prevalence of overweight and obesity among males than females in 2002, 2012, and 2018 [45], which was consistent with the findings of our study.

The incidence rate of thyroid cancer in urban areas was significantly higher than in rural areas. Studies have shown that stress and irritability may increase thyroid cancer risk, whereas good sleep quality serves as a protective factor [49]. A cross-sectional study conducted in China showed that urban residents exhibited a significantly higher insomnia rate (19.73%) compared to rural residents (16.99%) [50]. In contrast, the incidence of thyroid cancer in rural areas increased at a faster rate. With the improvement of the New Rural Cooperative Medical System (NRCMS) and the enhancement of its coverage and benefits, the economic barriers to medical care for rural residents were greatly reduced. Previous studies have shown that the coverage rate of the NRCMS increased from 75.20% in 2004 to 94.19% [51].

Age analysis showed that the trend of thyroid cancer incidence rate was basically consistent nationwide, first increasing and then decreasing with age, indicating that the risk of thyroid cancer was highest in middle-aged and elderly populations. Childhood exposure to risk factors such as radiation has long-term cumulative effects manifested in middle and old age [46]. The prevalence of thyroid nodules increases with age, with approximately 18.1% shown to be malignant [52, 53].

The results of the period analysis showed that the risk of incidence rate was on the rise, advancements in diagnostic techniques (such as high-resolution ultrasound) and their widespread clinical application, which enabled the detection of previously occult thyroid cancers [54, 55]. The overall birth cohort analysis showed a downward trend. On the one hand, the improvement of diagnostic capabilities led to a higher early detection rate; on the other hand, subsequent birth cohorts generally have higher health awareness, and they may adopt healthier lifestyles or seek medical intervention earlier [56].

Spatial distribution showed that East China had the highest concentration of thyroid cancer in 2010, with the eastern coastal region having a significantly higher incidence rate of ultrasound screening than other regions due to its developed economy and abundant medical resources [57]. The area of spatial aggregation shifted northwards between 2010 and 2012, with the Northeast region identified as a high–high cluster in 2013 and 2016., a region where dietary habits are typically high in salt [58]. Studies have shown that diets high in salt and fat are positively associated with the risk of thyroid cancer [59]."

*6 Other Information
6.1 Ethics marked 'N/A' with no explanation./Clarify that ethics approval was not required as de-identified secondary registry data were used, or specify if a waiver was granted.*

**Response:** We sincerely thank you for this important comment. We have added the following text (Page 13, Line 205-210):

**" Ethics Statement**

Ethical approval was not required for this study as it constituted analysis of pre-existing, aggregated, and fully de-identified data from the national China Cancer Registry Annual Report. The data contained no patient identities, private information, or biological samples. The use of such data does not require ethics review or participant consent."

**Reviewer 3**
*1. The findings reported in the current study largely confirm trends reported in prior studies including the increasing incidence and urban–rural contrast. Therefore it is necessary to provide clarification of what new the study would be adding to the existing evidence.*

**Response:** We sincerely thank you for this insightful comment. The primary novel contribution of our study lies in moving beyond confirming these trends to unveiling the underlying spatiotemporal dynamics and population-specific mechanisms. By integrating age-period-cohort modeling with spatial autocorrelation analysis, we provide a multifaceted characterization of the epidemic. Key new evidence includes the identification of a distinct shift in spatial clusters from Eastern to Northeastern China, the incidence risk initially increased and then declined with advancing age while cohort effects showed a downward trend, and the uncovering of faster growth rates in rural areas and among males. These findings offer fresh insights for targeted resource allocation and etiological research, adding a critical layer of depth to the existing evidence base.

We have added (Page 4-5, Line 48-65):

**"**However, at present, there is a lack of comprehensive studies on the spatial and temporal distribution of thyroid cancer incidence in China. Some studies are limited to specific geographical areas or populations, lacking representativeness on a national scale [8]. Additionally, although other studies have described the overall trend of thyroid cancer incidence in China, they have not systematically revealed the regional clustering characteristics of thyroid cancer incidence from a national perspective [9-11].

Comprehensive understanding of the epidemic characteristics of diseases is a key prerequisite for establishing an effective prevention and control system. As an ecological study, based on 14–years of data from the China Cancer Registry Annual Report from 2008 to 2021, this study described the epidemic characteristics of thyroid cancer incidence and its age-period-cohort effects, and used spatial autocorrelation methods to describe the spatial distribution characteristics. These analyses will help to gain a deeper understanding of the temporal and spatial distribution of thyroid cancer in China and its epidemiological trends, identify key areas for precise prevention and public health intervention of thyroid cancer, provide data to support long-term prevention and treatment efforts for thyroid cancer, and offer a reference for other countries around the world."

*2. For thyroid cancer, the unavailability of histo-pathological classification is an important limitation as overdiagnosis is a well-documented issue for thyroid cancer. Therefore this needs an in-depth discussion.*

**Response:** We sincerely thank you for raising this important point. We have added the discussion.

(Page 20, Line 284-305):

" The number of new thyroid cancer cases continued to rise, a trend driven by multiple factors. With advancements in thyroid cancer diagnostic techniques, the early detection rate has significantly improved. Results from a Chinese cohort study showed that the early detection rate of thyroid cancer increased from 29.98% in 2016 to 56.87% in 2020 [40]. However, these technological advances may also lead to a certain degree of overdiagnosis, resulting in the earlier detection of asymptomatic and non-lethal small lesions in the thyroid [41]. To address this issue, the China Guidelines for the diagnosis and treatment of thyroid cancer (2022) have stringently restricted biopsy indications and introduced active surveillance with close follow-up for low-risk micro papillary carcinoma [42]. Previous studies showed that both the incidence and mortality of thyroid cancer in China exhibited an upward trend [43]. This key evidence suggested that the increase in thyroid cancer incidence could not be entirely attributed to overdiagnosis, and there might be other risk factors contributing to the rise. Obesity is a probable factor. Studies have shown that for every 5kg/m² increase in body mass index, the risk of thyroid cancer may rise by 6%–25% [44]. According to the Report on Chinese Residents' Nutrition and Chronic Disease Status (2020), the overweight rate among residents aged 18 and above was 34.3%, an increase of 4.2 percentage points compared with that in 2012 [45]. This was consistent with the trends we uncovered in our research. In addition, at low radiation doses (<0.2 Gray), a linear relationship was observed between thyroid radiation exposure in children and subsequent thyroid cancer risk. This risk may appear 5–10 years after exposure and persists for more than 45 years [46]."

(Page 23, Line 347-357):

" This study had several limitations. First, this study was an ecological study conducted at the population level and may be subject to ecological fallacy. This study utilized data from the China Cancer Registry Annual Report, which contained only the number of thyroid cancer cases without details on pathological subtypes, tumor size, or disease stage. The lack of such information made it impossible to differentiate trends between subtypes or to precisely evaluate the degree of overdiagnosis and its contribution to the increasing incidence, thereby limiting a more profound analysis of the trends. Furthermore, the latest data had a three-year delay, and the number of monitoring points has increased, which may lead to bias in the results. Finally, due to the lack of more variable information, we did not conduct any further analyses, including sensitivity analysis. ".

*3. The authors have made a strong causative correlation of various causative factors such as obesity, stress, radiation exposure, however this causality is not established through the study design. Kindly write them as probable causative factors.*

**Response:** We sincerely thank you for the valuable comments from you. We made rigorous revisions to the discussion section.

(Page 20-23, Line 284-346):

" The number of new thyroid cancer cases continued to rise, a trend driven by multiple factors. With advancements in thyroid cancer diagnostic techniques, the early detection rate has significantly improved. Results from a Chinese cohort study showed that the early detection rate of thyroid cancer increased from 29.98% in 2016 to 56.87% in 2020 [40]. However, these technological advances may also lead to a certain degree of overdiagnosis, resulting in the earlier detection of asymptomatic and non-lethal small lesions in the thyroid [41]. To address this issue, the China Guidelines for the diagnosis and treatment of thyroid cancer (2022) have stringently restricted biopsy indications and introduced active surveillance with close follow-up for low-risk micro papillary carcinoma [42]. Previous studies showed that both the incidence and mortality of thyroid cancer in China exhibited an upward trend [43]. This key evidence suggested that the increase in thyroid cancer incidence could not be entirely attributed to overdiagnosis, and there might be other risk factors contributing to the rise. Obesity is a probable factor. Studies have shown that for every 5kg/m² increase in body mass index, the risk of thyroid cancer may rise by 6%–25% [44]. According to the Report on Chinese Residents' Nutrition and Chronic Disease Status (2020), the overweight rate among residents aged 18 and above was 34.3%, an increase of 4.2 percentage points compared with that in 2012 [45]. This was consistent with the trends we uncovered in our research. In addition, at low radiation doses (<0.2 Gray), a linear relationship was observed between thyroid radiation exposure in children and subsequent thyroid cancer risk. This risk may appear 5–10 years after exposure and persists for more than 45 years [46].

Our study indicated that thyroid cancer exhibited distinct gender-related patterns: females had a higher overall incidence. Estrogen-activated ERα may mediate the growth and malignant progression of thyroid cancer [47]. Among female thyroid cancer patients diagnosed in China between 1998 and 2012, overdiagnosis accounted for approximately 87% of cases [48]. In contrast, the incidence of thyroid cancer in men increased at a faster rate. The Report on Chinese Residents' Nutrition and Chronic Disease Status indicated a consistent trend of higher prevalence of overweight and obesity among males than females in 2002, 2012, and 2018 [45], which was consistent with the findings of our study.

The incidence rate of thyroid cancer in urban areas was significantly higher than in rural areas. Studies have shown that stress and irritability may increase thyroid cancer risk, whereas good sleep quality serves as a protective factor [49]. A cross-sectional study conducted in China showed that urban residents exhibited a significantly higher insomnia rate (19.73%) compared to rural residents (16.99%) [50]. In contrast, the incidence of thyroid cancer in rural areas increased at a faster rate. With the improvement of the New Rural Cooperative Medical System (NRCMS) and the enhancement of its coverage and benefits, the economic barriers to medical care for rural residents were greatly reduced. Previous studies have shown that the coverage rate of the NRCMS increased from 75.20% in 2004 to 94.19% [51].

Age analysis showed that the trend of thyroid cancer incidence rate was basically consistent nationwide, first increasing and then decreasing with age, indicating that the risk of thyroid cancer was highest in middle-aged and elderly populations. Childhood exposure to risk factors such as radiation has long-term cumulative effects manifested in middle and old age [46]. The prevalence of thyroid nodules increases with age, with approximately 18.1% shown to be malignant [52, 53].

The results of the period analysis showed that the risk of incidence rate was on the rise, advancements in diagnostic techniques (such as high-resolution ultrasound) and their widespread clinical application, which enabled the detection of previously occult thyroid cancers [54, 55]. The overall birth cohort analysis showed a downward trend. On the one hand, the improvement of diagnostic capabilities led to a higher early detection rate; on the other hand, subsequent birth cohorts generally have higher health awareness, and they may adopt healthier lifestyles or seek medical intervention earlier [56].

Spatial distribution showed that East China had the highest concentration of thyroid cancer in 2010, with the eastern coastal region having a significantly higher incidence rate of ultrasound screening than other regions due to its developed economy and abundant medical resources [57]. The area of spatial aggregation shifted northwards between 2010 and 2012, with the Northeast region identified as a high–high cluster in 2013 and 2016., a region where dietary habits are typically high in salt [58]. Studies have shown that diets high in salt and fat are positively associated with the risk of thyroid cancer [59]."

*4. Need to add more customised recommendations which are very generic.*

**Response:** We sincerely appreciate your valuable comment regarding the need for more customized recommendations. We have added (Page 23-24, Line 359-375): "From 2005 to 2018, the incidence rate of thyroid cancer in China consistently increased. The northeastern region was identified as a high-incidence hotspot for thyroid cancer. To effectively reduce the risk of thyroid cancer, the national monitoring system should be strengthened, and a registration system for high-risk populations, such as childhood radiation exposure, overweight individuals in high-incidence areas, should be established, accompanied by regular follow-up monitoring. For radiation exposure management, strict standardized procedures should be enforced in medical settings. A stratified management strategy should be implemented: for females, emphasis should be placed on rational screening and mental health maintenance; for males, focus should be on promoting weight management. At the regional level, urban areas should strictly comply with clinical guidelines for standardized diagnosis and treatment requirements, rural areas should improve health awareness; in the Northeast region, dietary interventions should be implemented to reduce the intake of high-salt and high-fat foods. This layered prevention and control strategy based on population characteristics and regional differences will significantly enhance the targeting and effectiveness of thyroid cancer control efforts in China."

**Reviewer 4**

1. *The use of the phrase 'effect coeeficient' was new to me, so I searched on net, google scholar, pubmed etc, and hardly found its mention. Does it mean 'effect size', kindly clarify or mention references supporting such usage.*

**Response:** Thank you very much for your comment and careful reading of our manuscript. The effect coefficient represents the logarithmic form of relative risk, and their exponential transformation yields relative risk values. The effect coefficient in the text were converted to relative risks (RR) to enhance interpretability. We have revised (Page 17, Line 237-247):

"From 2005 to 2018, the age effect of thyroid cancer in China exhibited an upward trend followed by a decline at the national level. The highest incidence rate risk was found in the 50-54 age group, including nationwide (RR=6.41, 95%CI: 5.18, 7.91), males (RR=7.40, 95%CI: 5.17, 10.61), females (RR=6.08, 95%CI: 4.90, 7.54), urban (RR=6.53, 95%CI: 5.25, 8.12) and rural (RR=5.60, 95%CI: 4.12, 7.61) areas.

The period effect of thyroid cancer in China, both nationally and across genders and regions, demonstrated an increasing trend during 2005–2015.

The 1990-2020 birth cohort showed the highest incidence risk, including nationwide (RR=7.66, 95%CI: 5.72, 10.25), males (RR=13.39, 95%CI: 6.84, 26.24), females (RR=6.29, 95%CI: 4.50, 8.79), urban (RR=9.05, 95%CI: 5.98, 13.70) and rural (RR=7.83, 95%CI: 2.63, 23.24) areas.”

2. *The limitations regarding more access to diagnosis being a confounding factor is well stated, and yet the need to combat obesity and radiation have been stated as public health measures.*

**Response:** We sincerely thank you for the valuable comments from you. We made rigorous revisions to the discussion section (Page 20-21, Line 284-305):

" The number of new thyroid cancer cases continued to rise, a trend driven by multiple factors. With advancements in thyroid cancer diagnostic techniques, the early detection rate has significantly improved. Results from a Chinese cohort study showed that the early detection rate of thyroid cancer increased from 29.98% in 2016 to 56.87% in 2020 [40]. However, these technological advances may also lead to a certain degree of overdiagnosis, resulting in the earlier detection of asymptomatic and non-lethal small lesions in the thyroid [41]. To address this issue, the China Guidelines for the diagnosis and treatment of thyroid cancer (2022) have stringently restricted biopsy indications and introduced active surveillance with close follow-up for low-risk micro papillary carcinoma [42]. Previous studies showed that both the incidence and mortality of thyroid cancer in China exhibited an upward trend [43]. This key evidence suggested that the increase in thyroid cancer incidence could not be entirely attributed to overdiagnosis, and there might be other risk factors contributing to the rise. Obesity is a probable factor. Studies have shown that for every 5kg/m² increase in body mass index, the risk of thyroid cancer may rise by 6%–25% [44]. According to the Report on Chinese Residents' Nutrition and Chronic Disease Status (2020), the overweight rate among residents aged 18 and above was 34.3%, an increase of 4.2 percentage points compared with that in 2012 [45]. This was consistent with the trends we uncovered in our research. In addition, at low radiation doses (<0.2 Gray), a linear relationship was observed between thyroid radiation exposure in children and subsequent thyroid cancer risk. This risk may appear 5–10 years after exposure and persists for more than 45 years [46]."

3. *I have not found, or may be missed where to look for the original data from the cancer registry. Some clarification will help.*

**Response:** We sincerely thank you for your comment regarding the data source. The cancer incidence data used in this study were indeed obtained from the National Cancer Registry System of China. The specific dataset has been officially published in the China Cancer Registry Annual Report. The relevant data as a supplementary file with the present submission.
